# Electrodialysis Processes an Answer to Industrial Sustainability: Toward the Concept of Eco-Circular Economy?—A Review

**DOI:** 10.3390/membranes13020205

**Published:** 2023-02-07

**Authors:** Aurore Cournoyer, Laurent Bazinet

**Affiliations:** Department of Food Sciences, Laboratoire de Transformation Alimentaire et Procédés ÉlectroMembranaires (LTAPEM, Laboratory of Food Processing and ElectroMembrane Processes), Institute of Nutrition and Functional Foods (INAF), Dairy Research Center (STELA), Université Laval, Quebec, QC G1V 0A6, Canada

**Keywords:** electrodialysis, sustainable development, circular economy, eco-efficiency, reuse, wastewater, by-product

## Abstract

Wastewater and by-product treatments are substantial issues with consequences for our society, both in terms of environmental impacts and economic losses. With an overall global objective of sustainable development, it is essential to offer eco-efficient and circular solutions. Indeed, one of the major solutions to limit the use of new raw materials and the production of wastes is the transition toward a circular economy. Industries must find ways to close their production loops. Electrodialysis (ED) processes such as conventional ED, selective ED, ED with bipolar membranes, and ED with filtration membranes are processes that have demonstrated, in the past decades and recently, their potential and eco-efficiency. This review presents the most recent valorization opportunities among different industrial sectors (water, food, mining, chemistry, etc.) to manage waste or by-product resources through electrodialysis processes and to improve global industrial sustainability by moving toward circular processes. The limitations of existing studies are raised, especially concerning eco-efficiency. Indeed, electrodialysis processes can be optimized to decrease energy consumption and costs, and to increase efficiency; however, eco-efficiency scores should be determined to compare electrodialysis with conventional processes and support their advantages. The review shows the high potential of the different types of electrodialysis processes to treat wastewaters and liquid by-products in order to add value or to generate new raw materials. It also highlights the strong interest in using eco-efficient processes within a circular economy. The ideal scenario for sustainable development would be to make a transition toward an eco-circular economy.

## 1. Introduction

The concept of sustainable development is common; nevertheless, it is still evolving and is a major concern in today’s global environmental, social, and economic context. The idea to establish more sustainable systems to address major environmental issues took place after the 1972 United Nations Conference on the Environment in Stockholm. Particularly, they created the United Nations Environment Program (UNEP) [1]. Nowadays, 50 years after the first environment conference, the urge for “bold actions” on the triple planetary crisis of climate change, pollution, and biodiversity loss is still major [2]. In 2015, the United Nations settled 17 sustainable development goals to achieve by 2030. Hence, the importance to move toward a circular economy and eco-efficient processes occurred in this context. Indeed, they represent strategies to implement global sustainability.

Instead of considering wastes and by-products as an accumulated burden for society, they must be seen as opportunities to create new products and become raw materials for new processes or applications. However, the use of wastes and by-products involves treatment and transformation, which should be performed in a more eco-efficient way to balance the environmental and economic benefits. Wastewater and liquid by-products are problematic in different industrial fields such as the chemical, metallurgical, petroleum, agri-food, and leather industries. Indeed, large volumes of liquid waste produced by these industries, with high salinity and high mineral and/or organic content, have impacts on ecosystems and the salinity of soils, which cause the deaths of aquatic and other species [3,4]. For example, in 2017, the volume of water discharge in mineral extraction and thermal-electric power generation industries represented about 24 billion cubic meters in Canada [5]. In the same way, blood collected from slaughterhouses represents substantial volumes. For example, only for pork in Canada, approximately 68 million litres are recovered annually [6]. The increasing complexity of industrial effluents and their known consequences on the environment force academics, industrials, and politicians to provide concrete and worldwide solutions.

Several treatments are used to handle these waters or by-products such as biological, chemical, or physicochemical methods, depending on the objectives. Biological methods are based on the ability of microorganisms to biodegrade the pollutants, but high concentrations of salts or toxic compounds might inhibit their growth or the production of certain metabolites of interest, such as hydrocarbons in petrochemical effluents [7]. Chemical methods refer to chemical oxidation to produce more stable or degradable molecules. However, at the end of the process, it was shown that intermediate compounds are not always degradable, and the method must be combined [8]. Finally, physicochemical methods consist of phase separation such as decantation, absorption on membranes, or coagulation–flocculation [9]. In this case, all fractions generated need to be usable to not generate new wastes. It was demonstrated that coupling processes were an alternative to overcome some of these limitations [8]. Recently, electrodialysis (ED), an electrochemical method, showed real potential in the reuse or valorization of by-products and wastewater treatments in an eco-efficient way [3,10,11]. Indeed, this electromembrane process is a very versatile technology with diversity in the choice of membranes, electrochemical phenomena, current conditions, and configurations and has the ability to be coupled with other methods. Electrodialysis technologies have demonstrated the capability to achieve goals of sustainable development in a more eco-efficient way and in a circular economy framework.

This review presents the most recent valorization opportunities among different industrial sectors (water, food, mining, chemistry, etc.) to manage waste or by-product resources through electrodialysis processes and to improve global industrial sustainability. In the first section, the concepts of sustainable development, eco-efficiency, and circular economy are described. Then, in the second section, the main types of electrodialysis processes and recent innovations are summarized. Finally, sustainable electrodialysis strategies separated according to their general objective are presented: (1) increase eco-efficiency; (2) include the process in a circular economy framework; or (3) combine both objectives—an eco-efficient process in a circular economy.

## 2. Definitions

### 2.1. Sustainable Development

**Figure 1:**
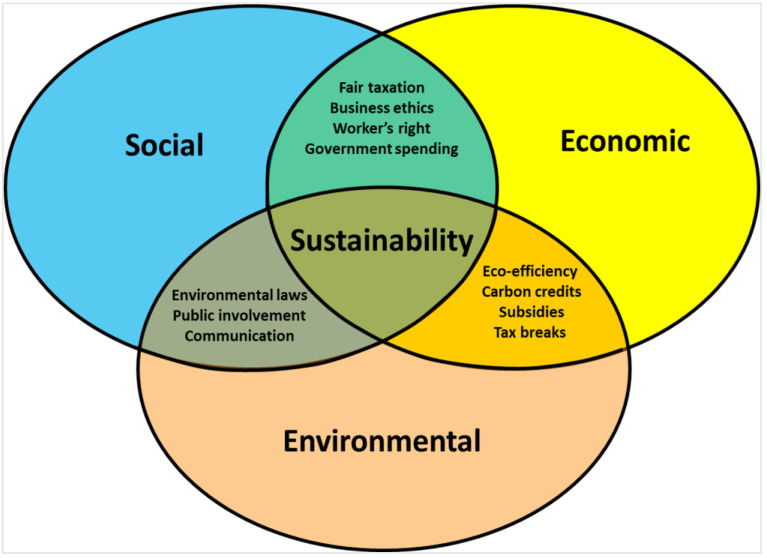
Interconnection of the 3 sustainable development pillars to reach sustainability (adapted with permission from Mensah (2019) [12]).

Sustainable development (SD) is a broad concept that encompasses three principal components: the environment, society, and the economy, working together to produce sustainable benefits. The balance between the three pillars is prevalent because of their interconnection (Figure 1). SD intends to achieve social improvements, environmental equilibrium, and economic development [12]. Every decision and action must reach the needs of the three interrelated concepts [13]. It is interesting to mention the proposition to consider sustainability as an established and functioning system, and SD as the entire process to achieve this state [14]. Sustainability should be a staple topic in politics, at the same level as justice, democracy, and liberty.

Moreover, SD is about long-term changes that positively impact the population of the present and the populations of the future. It is not about sporadic improvements, but prolonged ones for future generations. As the United Nations World Commission on Environment and Development declared in their report “Our common future” in 1987, sustainable development is a “development that meets the needs of the present without compromising the ability of future generations to meet their own needs.” [15]. The commitment of the people and efforts from various levels of society are needed to turn the concepts into actions [12].

### 2.2. Eco-Efficiency

Sustainable development concerns have increasingly gained importance among different worldwide organizations and their stakeholders. Consequently, eco-efficiency (EE) has become a consistent tool in the transition to sustainability. EE indicators or scores are useful to compare studies and to take decisions [16]. The prefix “eco” stands for both ecological and economic performance, but in a broader sense, eco-efficiency also covers the social dimension of sustainable development [16,17,18].

Hence, EE allows us to measure sustainability with the aim of reducing the resources needed and the impact on the environment while maintaining or increasing product value. This was intended to be used primarily by companies. This concept was introduced for the first time during the United Nations conference on environment and development in 1992, in Rio de Janeiro, by the World Business Council for Sustainable Development (WBSCD). This association, composed of 120 companies from different countries, defined EE as: “The delivery of competitively-priced goods and services that satisfy human needs and bring life cycle, to a level at least in line with the earth’s estimated carrying capacity” [17]. The aim was to allow companies to increase their contribution to an emerging sustainable society while remaining competitive [19]. One of the main delicate parts to implement this strategy was the definition of the EE score (Equation (1)) [20]. Indeed, EE is a relative concept, and indicators are needed to perform comparisons. At first, companies used internal evaluation systems for EE, then internal indicators [21], and, finally, the International Organization for Standardization (ISO) proposed, in 2012, a standardized method: ISO 14045 [22]. Eco-efficiency is a key concept of proper indicators that can help a company to achieve more sustainable development [23].

(1) $$Eco\text{-}efficiency\;score=\frac{Value\;of\;the\;product}{Sum\;of\;environmental\;impacts}$$

In most cases, EE studies define the product value (the numerator in Equation (1)) by monetary units, such as the potential benefits [24,25], or by desired outputs [26,27]. Generally, the economic value-added is calculated as the value of sales minus the cost of goods [28], a life cycle cost [29], or net sales activities [30]. According to ISO 14044, “product system value may encompass different value aspects, including functional, monetary, aesthetic, etc.” [31]. To the best of our knowledge, Chaudron et al. [32] (functional nutrient content, taste, and level of abatement of harmful substances) and Faucher et al. [33] (polyphenol content and amount of organic acid removed) were the first to carry out a multi-criteria assessment of value from the consumer’s perspective, in comparison to EE studies using LCA methodology as the denominator (Table 1). Very recently, Houssard et al. [34], on Greek yogurt production, proposed a multidimensional value assessment model, taking into account the financial, socio-economic, and functional components of value typically created by a food value chain (Table 1). It is important to highlight that the ISO 14045 standard does not impose any restrictions on the definition of value. Nevertheless, it has been demonstrated that the use of functional value indicators makes it possible to overcome the limitations related to the use of monetary indicators (e.g., price is not always a quality indicator for the consumer, information regarding monetary value is not always known in the early stages of product development or when scaling up, etc.) [32,35]. Hence, by using different value indicators, the decision-maker could target their decision on important product functionalities for which the consumer is looking, beyond the sales price. This approach is complementary to traditional monetary-based approaches and is useful to design cleaner production technologies that minimize environmental impacts across the whole life cycle whilst providing higher value to consumers [32].

**Figure 2:**
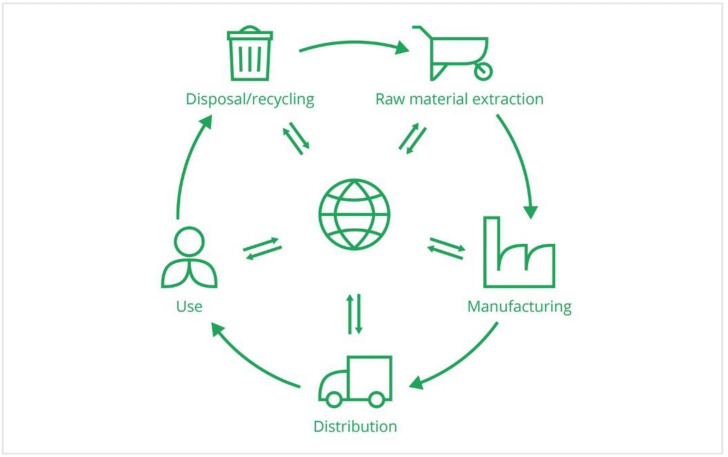
Life-cycle assessment (LCA) diagram (with permission from Golsteijn L. [38]).

Concerning the determination of a denominator, the “sum of the environmental impacts”, it must be based on a life-cycle assessment (LCA). It is a standardized, science-based, and quantitative method. Indeed, a norm to realize LCA, ISO 14044, and one to describe the principles, evaluation tools, and more, ISO 14040, were created to guide the evaluation of the environmental impacts of a functional unit [21]. The functional unit refers to the product, service, or system whose impacts are calculated using an LCA methodology (e.g., one metric ton of cheese produced, one cubic meter of water desalinated, etc.) [36]. Consequently, the choice of the functional unit impacts greatly on the LCA’s results, and it is important to be careful when the results of LCAs using different functional units are compared. Generally speaking, the LCA methodology evaluates the environmental impacts of processes (products, services, or systems) during their entire life cycle (Figure 2). All the stages of a product’s life are analyzed, from raw material extraction, through materials processing, manufacture, and distribution, to the use of the product [37].

Although a rigorous LCA relying on multiple indicators such as climate change, human health, consumption of resources, or ecosystem quality is recommended to obtain reliable long-term data, EE analysis, according to the ISO standard and the LCA to be carried out, can be a tedious task to undertake. Indeed, collecting data for the inventory to be carried out during the LCA is a colossal task that requires a lot of time and resources. It can also be difficult to access this data for industrial processes where it is often kept confidential. However, it is possible to use a simplified approach to assess eco-efficiency that uses environmental impact indicators instead of environmental impact scores determined by an LCA [39,40,41] (Table 1). These indicators, determined by an analysis of the process, are linked to inputs or outputs contributing significantly to the environmental impact (e.g., energy consumption, water consumption, etc.). Without being as precise as the results obtained by an LCA, this method provides an overall idea of the environmental repercussions associated with the process. As a result, it is possible to calculate eco-efficiency scores that will make it possible to identify general information, discriminate between certain options, guide decisions, etc., much like what can be achieved when interpreting the results of an eco-efficiency analysis carried out according to the ISO standard [35].

**Table 1:** Recently proposed eco-efficiency scores.

Basis of the Eco-Efficiency Score	Product	Eco-Efficiency Score	Components of the Eco- Efficiency Score	Rationals and Parameters	Ref.
Health benefits	Cranberry juice	$EE=\frac{Value\;of\;the\;product}{Sum\;of\;environmental\;impacts}$	*Value of the* *product =* ${price}_{cranberry\;juice}+{price}_{polyphenol\;compounds}$ *Sum of environmental impacts:* *based on an LCA*	Cranberry juice’s health benefits are associated with its high polyphenol content. Hence, the value was related to the polyphenol content of the product and calculated as the sum of the price of 1000 kg of cranberry juice and the price of the polyphenolic compounds present in the deacidified juice. The life-cycle assessment was conducted according to ISO 14044.	[33]
		$EE=\frac{Functional\;value}{Environmental\;impact\;cost}$	*Functional value = content of functional nutrients* *Environmental impact cost:* *based on an LCA*	Polyphenol content was selected as a functional value parameter due to its beneficial health effects. The life-cycle assessment was conducted according to ISO 14044.	[32]
		$EE=\frac{Functional\;value}{Environmental\;impact\;cost}$	*Functional value = abatement of harmful substances* *Environmental impact cost:* *based on an LCA*	The concentration of organic acids may induce gastrointestinal disturbances. The percentage of removed harmful acid content was chosen as a functional value parameter. The life-cycle assessment was conducted according to ISO 14044.	[32]
	Sweet whey and whey protein concentrate (WPC)	$EE=\frac{Value\;of\;the\;product}{Environmental\;impacts}$	$Value\;of\;the\;product=\frac{g\;of\;PLs\;recovered}{g\;of\;crude\;protein\;treated}$ *Environmental impacts:* *Volume of water used and effluent produced*	Phospholipids (PLs) have a positive impact on the brain, aging, and neurodegenerative diseases, as well as on cell growth and the prevention of colon cancer. The ratio of PLs quantity recovered (in g) per quantity of crude protein treated (in g) was chosen as a value parameter to compare sweet whey and WPC on the same basis. Volume of water and effluents involved in the process (in L) as an environmental parameter.	[42]
		$EE=\frac{Value\;of\;the\;product}{Environmental\;impacts}$	$Value\;of\;the\;product\;=\frac{g\;of\;PLs\;recovered}{g\;of\;crude\;protein\;treated}$ *Environmental impacts:* *Volume of water used and effluent produced*	As high PL content is desired in the final fraction obtained by precipitation, the ratio of PLs in the precipitated fraction over protein recovered in this same fraction was used as a value parameter. Volume of water and effluents involved in the process (in L) as an environmental parameter.	[42]
Health benefits	Sweet whey and whey protein concentrate (WPC)	$EE=\frac{Value\;of\;the\;product}{Environmental\;impacts}$	$Value\;of\;the\;product=\frac{g\;of\;PLs\;recovered}{g\;of\;crude\;protein\;treated}\times \frac{g\;of\;PLs\;recovered}{g\;of\;protein\;in\;the\;PL\;fraction}$ *Environmental impacts:* *Volume of water used and effluent produced*	Value product as a combination of both previous ratios to take into account PL yield in terms of protein treated and the purity of the final fraction. Volume of water and effluents involved in the process (in L) as an environmental parameter.	[42]
	Whey protein hydrolysate (WPH)	$EE=\frac{ACE-inhibiting\;value}{Environmental\;impact\;cost}$	$ACE-inhibiting\;value=\frac{{IC}_{50\;ACE\;Captopril}\times {price}_{Captopril}\times m_{fraction\;1000\;runs}}{{IC}_{50\;ACE\;fraction}}$ Environmental impact cost = Energy cost + Chemical cost	IC_50 ACE_ fraction was normalized using the IC_50 ACE_ of Captopril as an ACE inhibitor standard. Factoring the market cost per kg of this standard and the mass of fraction recovered after 1000 EDUF runs. Environmental impact cost was calculated by summing the energy cost and the chemical cost for 1000 EDUF runs.	[40]
Process added-value	Beta-lactoglobulin	$EE=\frac{DH\;improvement}{Energy\;consumption}$	$DH\;improvement=\frac{DH\;pretreated\;protein}{DH\;native\;protein}$ ×100 $Energy\;consumption\;=Q+W+\frac{\left(N\times Pi\right)}{m}$	Degree of hydrolysis (DH) was determined by the ophthaladehyde (OPA) method. Energy consumption (EC) was the specific energy input in MJ/kg for treatments of protein solution with Q, the total energy input, to pasteurize 1 kg of β-lg solution (MJ/kg); W (0.1 MJ/kg), the total work input to the electrical motors to pasteurize 1 kg milk; N, the number of pulses; m, the mass (kg) of the treated protein solution; and Pi, the energy of one electric pulse.	[41]
		$EE=\frac{\Delta Cpeptide}{Energy\;consumption}$	*∆C_peptide_ = 1 × 10^6^*× ($\frac{A_{214}\;in\;pretreated-A214\;in\;native}{\varepsilon_{214}\times l{\times V}_{inj}{\times K}_{cell}}$) ×*f* $Energy\;consumption\;=Q+W+\frac{\left(N\times Pi\right)}{m}$	ΔCpeptide (in μM) represents the improvement in the relative concentration of bioactive peptides identified in pretreated β-lg hydrolysates in relation to their concentration in native ones. Energy consumption was the specific energy input in MJ/kg for treatments of protein solution with Q, the total energy input to pasteurize 1 kg of β-lg solution (MJ/kg); W (0.1 MJ/kg), the total work input to the electrical motors to pasteurize 1 kg milk; N, the number of pulses; m, the mass (kg) of the treated protein solution; and Pi, the energy of one electric pulse.	[41]
	Cranberry juice Cranberry juice	$EE=\frac{Value\;of\;the\;product}{Sum\;of\;environmental\;impacts}$	*Value of the product =* ${price}_{cranberry\;juice}+{price}_{citric\;acid}+{price}_{malic\;acid}$ *Sum of environmental impacts:* *based on an LCA*	The value of the product was linked to the amount of organic acids removed (citric and malic) and available for other industrial applications; the value was calculated as the sum of the price of 1000 kg of cranberry juice and the price of the amounts of citric and malic acids removed and usable after the deacidification treatment. The life-cycle assessment was conducted according to ISO 14044.	[33]
		$EE=\frac{Value\;of\;the\;product}{Sum\;of\;environmental\;impacts}$	*Value of the product =* ${adjusted\;price}_{cranberry\;juice}$ *Sum of environmental impacts:* *based on an LCA*	The value of the product was calculated as the price of cranberry juice with an adjustment when quinic acid was lost during the deacidification treatment: if there was no significant loss of quinic acid in the deacidified juice, the juice was considered non-tampered and its value was 100% of the price. If there was a significant loss of quinic acid, the juice was considered tampered and its value was 80% of the selling price of 1000 kg of cranberry juice (loss of 20% of the regular selling price). The life-cycle assessment was conducted according to ISO 14044.	[33]
		$EE=\frac{Value\;of\;the\;product}{Sum\;of\;environmental\;impacts}$	*Value of the product =* ${adjusted\;price}_{cranberry\;juice}+{price}_{polyphenol\;compounds}+{price}_{citric\;acid}+{price}_{malic\;acid}$ *Sum of environmental impacts:* *based on an LCA*	The value of the deacidified juice was the sum of the price of 1000 kg of commercially available cranberry juice with price adjustment considering significant loss or not of quinic acid, the price of the polyphenolic compounds present in the deacidified juice, and the price of the citric and malic acids removed and usable. The life-cycle assessment was conducted according to ISO 14044.	[33]
	Mealworm protein	$EE=\frac{Value\;of\;the\;product}{Global\;warming\;potential}$	*Value of the product = g Protein x kg product* *Global warming potential = kg CO_2_ éq x kg product*	The protein content of the mealworm extract (or other protein sources) was considered the product value. Global warming potential represents the environmental impact.	[43]
	Whey protein hydrolysate (WPH)	$EE=\frac{Environmental\;impact\;cost}{\%recovery}$	Environmental impact cost = Energy cost + Chemical cost $\%recovery=\frac{AUC_{bioactive\;peptides}}{AUC_{WPH}}$	The environmental impact cost was calculated based on the energy cost and the chemical cost of 1000 EDUF runs. The recovery of bioactive peptides (in %) was expressed with the area under the curve of bioactive peptide (AUC_bioactive peptides_) and AUC_WPH_, added to the areas under the curve (LC–UV data) of individual bioactive peptides in the recovery fraction at t_180 min_ and in the initial WPH at t_0min_, respectively.	[39]
Consumers/stakeholders’ Criteria	Cranberry juice	$EE=\frac{Functional\;value}{Environmental\;impact\;cost}$	*Functional value = Taste* *Environmental impact cost:* *based on an LCA*	The taste, particularly the acidic taste, stops some clients from buying cranberry juice. Hence, taste was selected as a functional value parameter. Taste was determined following an organoleptic test. The life-cycle assessment was conducted according to ISO 14044.	[32]
		$EE=\frac{Functional\;value}{Environmental\;impact\;cost}$	*Functional value = abatement of harmful substances* *Environmental impact cost:* *based on an LCA*	The concentration of organic acid impacts the acidic taste of the juice, which stops some clients from buying cranberry juice. The percentage of removed harmful acid content was chosen as a functional value parameter. The life-cycle assessment was conducted according to ISO 14044.	[32]
	Greek yogurt	$EE=\frac{Value\;of\;the\;product}{Environmental\;impact}$	*Value of the product = Financial profit + Socio-economic value + Functional value* *Environmental impact:* *based on an LCA*	*Financial profit* is the net income from sales after the deduction of all costs related to production, including material costs, capital costs, labour costs, and taxes. *Socio-economic value* is composed of the socio-economic value measured by the gross value added (GVA) at the territory level and the socio-economic value measured by the total gross value added (Total GVA) at the society level. *Functional value* refers to nutritional attributes, health benefits, and sensory characteristics (texture, taste, flavour, etc.) and also encompasses packaging functionalities (shelf life, practicality, lightness, robustness, aesthetics, etc.). Calcium content, probiotic concentration, and typical flavour, representative of the main functions sought by Greek yogurt consumers, were selected as functional indicators. The life-cycle assessment was conducted according to ISO 14040 and 14044.	[34]

EE: eco-efficiency score; LCA: life-cycle assessment.

### 2.3. Circular Economy

**Figure 3:**
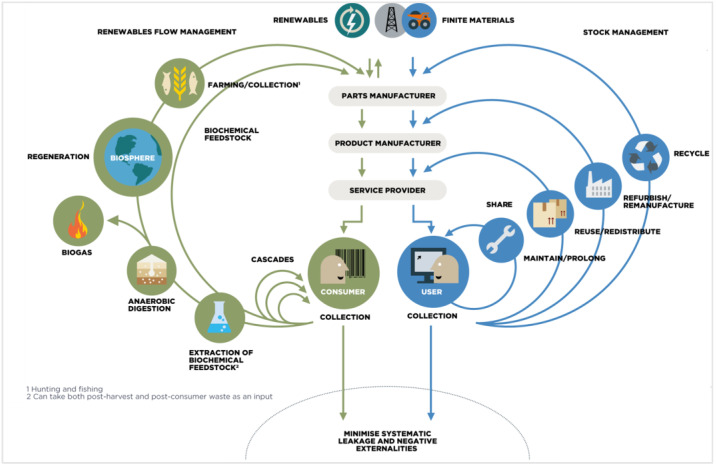
Butterfly diagram of circular economy (with permission from the Ellen MacArthur Foundation (2019) [46].

The concept of circular economy (CE) is still emerging. This strategy has been defined several times in the literature, but more recently by the Ellen MacArthur Foundation [44]. The aim is to minimize contaminants and eliminate pollution using closed loop(s). Products must have a longer shelf-life and a new purpose at the end by becoming the raw material of a new process [45]. Particularly, the product must be able to return safely to the earth. Waste, the use of virgin natural resources, and associated pollution are reduced to a minimum [45]. According to the Foundation, a circular economy is divided into three main principles: (1) eliminate waste and pollution; (2) circulate products and materials at their highest value, which means preserving the product’s entirety to retain the maximum value; and (3) regenerate nature [45]. CE is applicable as much to biological as to technological materials. A butterfly diagram was designed to illustrate the large concept of CE (Figure 3). Indeed, two main cycles are involved. First, the technical cycle represents products and materials that circulate in the economy thanks to recycling, remanufacturing, repairing, and reusing. Then, the biological cycle speaks for biodegradable materials, which aim to return to earth and rebuild natural capital [46].

A highlighted difference from SD is the beneficiaries. It was reported that SD impacts society, environment, and economy at large, and the problems involved are open-ended. On another side, the main beneficiaries of the implementation of a new circular economy system are economic actors. Then, at a smaller scale, the environment and society benefit from positive impacts through the reduction in resources needed, the reduction in pollution, and the creation of jobs [47].

The relationship between sustainable development, eco-efficiency, and circular economy is becoming clearer. EE is a way to optimize a circular system, which is fundamental to achieving sustainability. Nevertheless, it is important to mention that different views exist in the literature concerning the understanding of the relationships between the different concepts [47]. On one hand, the Ellen MacArthur Foundation has proposed well-known explanations of these concepts, considering CE and SD as dependent, with a strong conditional relationship [48]. Hence, SD needs CE to fulfill its objectives, and CE is always allowing a step toward SD. On the other hand, circular strategies were also described as one option for sustainable business models, among others, to reach sustainability [49]. Indeed, other possibilities described are increased efficiency and dematerialization, which represents a reduction in energy consumption and the need for raw materials or natural resources to produce and exchange products [47,50].

## 3. Electromembrane Processes: General Aspects

Electrodialysis (ED) is a separation method using membranes, based on electric potential difference, to separate organic or mineral-ionized species and, generally speaking, charged molecules. The main types of ED systems that can be used to treat wastewater and different by-products are presented below. More specific information concerning all the different systems available has been reported in the recent reviews or book chapters of Koseoglu-Imer et al. (2018) [51], Scarazzato et al. (2020) [52], Bazinet et al. (2020) [11], Gurreri et al. (2020) [53], Mir and Bicer (2021) [54] and Arana Juve et al. (2022) [55].

### 3.1. Electrodialysis with Ion-Exchange Membranes

**Figure 4:**
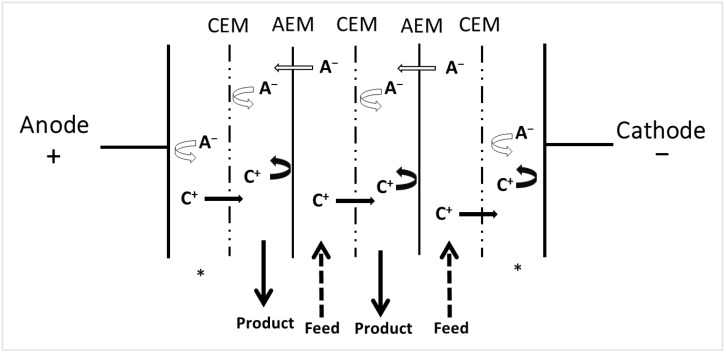
Schematic conventional electrodialysis configuration. * Electrode rinsing solution; CEM: cation-exchange membrane; AEM: anion-exchange membrane; C^+^: cation; A^−^: anion.

ED with ion-exchange membranes (IEM) is considered the conventional ED method. The configuration is a stacking of cation-exchange membranes (CEM) and anion-exchange membranes (AEM) (Figure 4). An electrolyte solution is pumped between the membranes under an electrical potential initiated between electrodes. After treatment, not considering the electrode rinsing solution, two products are obtained: a feed diluted in ions, the diluate, and a product concentrated in migrated ions, the concentrate. The earliest work on electrodialysis was reported in 1890 when Maigrot and Sabates [56] patented a three-compartment cell. In this simplest form of electrodialysis, the membranes were used as physical barriers to prevent the mixing of electrolysis products [57]. The first ED desalination plant was built by Ionics in 1954 for Aramco (Saudi Arabia) [58], and a few years later, ED units for brackish water desalination were built in South Africa [59]. More recently, it was studied for food industry applications such as the demineralization of whey [60], the stabilization of wine [61], the deacidification of fruit juices [62], etc. Several reviews or book chapters that classify ED applications are available [58,63,64].

Based on conventional ED, selective ED or selectrodialysis (SED) was developed in 2012, by Zhang et al. [65], to handle wastewater. Nutrients and valuable ions are valorized using this divalent and monovalent ion fractionation technology. The SED configuration is a conventional ED configuration (Figure 4) with the addition of one or more permselective membranes between the AEM and the CEM: a monovalent selective anion- or cation-exchange membrane. SED involves three different streams: the desalinated feed, the product containing the divalent ions, and the brine, with concentrated monovalent ions. Thus, the purpose is to remove specific monovalent ions while specific divalent ions are concentrated in the product fraction [65].

### 3.2. Electrodialysis with Bipolar Membranes

**Figure 5:**
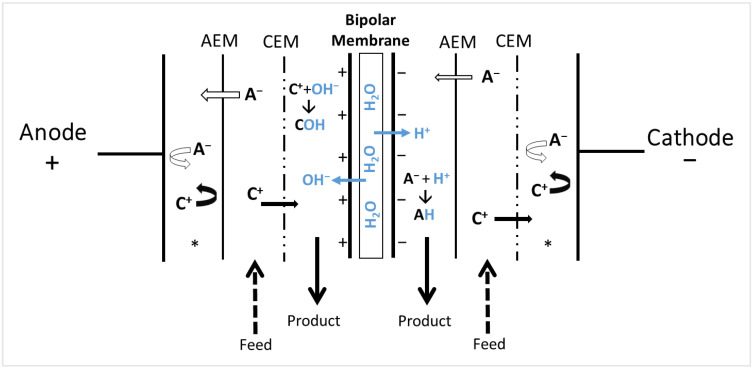
Schematic EDBM configuration and water dissociation phenomenon at the interfaces of a bipolar membrane [53,68]. * Electrode rinsing solution; A^−^: anion or negatively charged molecule (e.g., organic acid anionic form); C^+^: cation or positively charged molecule (e.g., peptide or protein, positively charged when the pH is under their isoelectric point); CEM: cation-exchange membrane; AEM: anion-exchange membrane.

The bipolar membrane is a very specific membrane composed of three distinct parts: a cation-exchange layer, an anion-exchange layer, and an intermediate hydrophilic layer between the CEM and AEM layers (Figure 5). When the membrane is exposed to an electric current, water dissociation takes place and generates H^+^ and OH^-^, respectively, through cationic and anionic layers [66,67]. A different stack of membranes is possible to form electrodialysis with a bipolar membrane (EDBM) cell. The cell could be composed of six compartments, with one AEM facing the cation-exchange layer of the BM, followed by a CEM, and one CEM facing the anion-exchange layer of the BM, followed by an AEM (Figure 5). The solution circulating in the H^+^-released compartment is acidified and, similarly, the other solution is basified. H^+^ and OH^-^ can also react with the ions available to form organic acids or bases, for example [68]. EDBM is particularly useful for the production of acids and bases from corresponding salt solutions, which is beneficial for the protection of the environment and the management of waste or by-products from a wide variety of industries [53,69]. The first commercial application of EDBM happened in 1986 [70] and aimed to regenerate acids from metallurgical industry liquors or recover acids and bases from salt streams [71,72]. In food processing [73], the main applications were demonstrated for the inhibition of enzymatic browning in juices [74], the deacidification of fruit juices [33], the purification and fractionation of proteins [64], and, very recently, the separation of phospholipids [42].

### 3.3. Electrodialysis with Filtration Membranes

**Figure 6:**
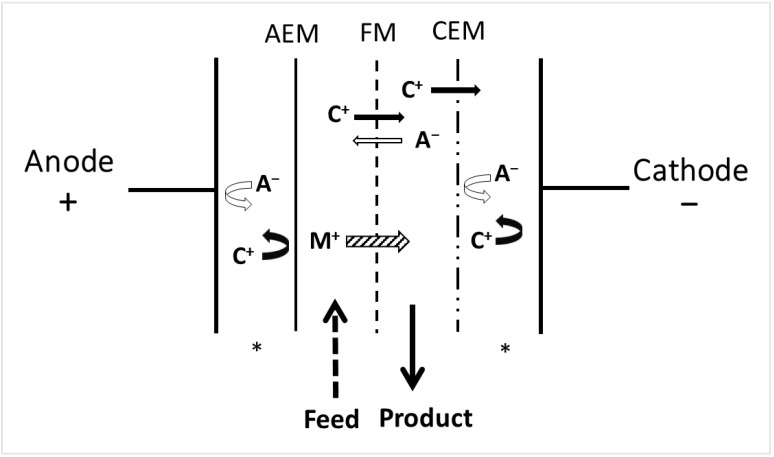
Schematic EDFM cationic configuration. * Electrode rinsing solution; A^−^: anions; C^+^: cations; M^+^: positively charged molecule of interest; CEM: cation-exchange membrane; AEM: anion-exchange membrane; FM: filtration membrane.

In 2005, Electrodialysis with filtration membranes (EDFM), a hybrid technology, was developed and patented [75]. EDFM is a selective separation process due to the replacement of some ion-exchange membranes in the stack by filtration membranes: microfiltration, ultrafiltration, or nanofiltration. Depending on the molecular weight cut-off (MWCO) of the membrane, specific charged molecules migrate or not through the membrane. This technique is very selective thanks to the criteria of separation: charge and molecular weight [11,68]. Different configurations are available depending on the charge and molecular weight of the compounds of interest. Cationic (Figure 6) or anionic (in this case, the polarity is changed) configurations to recover, respectively, positively or negatively charged molecules can be selected. Configurations with two recovery compartments (the simultaneous recovery of anionic and cationic molecules) or multiple recovery compartments (three anionic or cationic compartments created by stacking filtration membranes with different molecular weight cut-offs, as well as two cationic and two anionic recovery compartments in the same stack) [76] were also demonstrated to be effective [77,78]. Different parameters can be selected to influence the selectivity and thus recover specific compounds: the pH of the feed, electrical field strength, conductivity, membrane physicochemical characteristics, the MWCO of the membrane, the mode of current, etc.) [11,39,75,76,77,78,79,80]. Applications in several fields have been demonstrated, such as the separation of bioactive peptide fractions [77,78,79], the improvement of protein hydrolysate bioactivity [80], the selective recovery of monovalent ions [81], the separation of antibiotics from wastewater [82], the simultaneous hydrolysis and separation of peptides [83,84], and also the separation or concentration of protein [85], chitosan oligomers [86], and phenolic compounds [87]. Electrodialysis with ultrafiltration membranes (EDUF), a specific configuration of EDFM (Figure 6) using an ultrafiltration membrane as an FM, demonstrated high feasibility for separating charged bioactive peptides at laboratory [75,76,77,78,79,80] and semi-industrial [40] scales. Moreover, electrodialysis with a nanofiltration membrane (EDNF) showed promising results for improving the demineralization rate [81,88] and acid recovery [88]. The technology was tested for the separation of cations and anions and demonstrated a high potential for applications in the chloralkali industry [88,89,90] or for desalinization [81,88].

The EDFM methods and configurations allow the recovery of an important diversity of compounds. Different methods are also available, such as pressure-driven membrane technologies, but electromembrane processes have proven their advantages for charged compounds or when the final bioactivities are a main concern. Indeed, for bioactive peptides, EDUF allows the reliable, selective, and cost-effective separation of specific bioactive peptide fractions (0.25–0.50 US $/g of bioactive peptide estimated for a 10 m^2^ membrane surface) from complex mixtures when compared to conventional methods such as chromatography or pressure-driven separation [91,92].

## 4. Electrodialysis and Eco-Efficiency

As seen earlier, in the actual context of sustainability, EE is a quantitative strategy to improve processes. To improve the EE of existing electrodialysis technologies, innovative processes, coupling processes, or the optimization of parameters are studied in different fields of application.

### 4.1. Seawater and Municipal Wastewater

Seawater reverse osmosis (RO) is the main technology used for the desalination of seawater. Despite global attention, several RO limits remain, such as final water quality, recovery factor, cost, and brine disposal [93], which are major issues. Indeed, the recovery factor is limited to 50% by the osmotic phenomenon [94]. Hence, a large volume of brine is produced and has negative impacts on the environment, such as damage to water ecosystems. In this context, reverse electrodialysis (RED), conventional electrodialysis, and electrodialysis metathesis (EDM) were proposed as answers to improve seawater RO processes.

Reverse electrodialysis (RED) showed an interesting capacity when integrated into an RO-membrane distillation–RED desalination system [95]. This membrane process is based on a conventional ED configuration, stacking CEMs and AEMs. Unlike in ED, the driving force comes from the ion’s potential differences between “High Concentration” and “Low Concentration” compartments, which creates an electricity gradient instead of applying an external potential difference. RED is known for its renewable energy generation through the flux of ions that reaches electrodes where redox reactions allow the continuous generation of electricity [95]. The most optimized hybrid system demonstrated an improvement in the energy efficiency of the global process, with up to 17% reduction in electrical energy consumption and 8% in specific energy consumption (thermal and electrical energy per volume of desalted water) compared to RO alone [94]. The study demonstrated a new step forward in the implementation of zero-liquid discharge systems and low-energy desalination thanks to the ability to produce renewable energy and drinkable water [94]. No EE score was determined through the study to compare both methods.

Conventional ED also demonstrated the capacity to treat concentrated brine obtained after desalination by RO [96]. ED allowed the production, from RO brine, of fresh water with a water recovery rate of 67.8% and a desalination rate of 72.5%. Operation parameters such as current density, operation mode, type of membrane, and initial brine concentration were compared in this study to obtain the more efficient method: an increase in the membrane’s ion-exchange capacity (IEC) had the most positive impact on the concentration and current efficiencies [96]. The most adequate method had an IEC of 1.25 mEq.g^−1^ for AEM and 1.62 mEq.g^−1^ for CEM, compared to the less effective method, which had an IEC of 1.07 mEq.g^−1^ and 0.74 mEq.g^−1^, respectively. Indeed, a high IEC involves a higher perm-selectivity of the membrane and a lesser co-ion transportation phenomenon, which involves better concentration and current efficiencies. For the same current density, initial mass concentration, and volume ratio, the highest IEC membranes allowed 15% higher desalination compared to the others [96]. The study compared ED efficiency through different ED parameters, but no global EE scores were calculated to really compare the different parameters or conditions used. Improvements in the concentration and current efficiency might indeed improve the EE score, but it must be investigated and confirmed.

Electrodialysis metathesis (EDM) was also used for desalination and the efficiency of the process was affected mainly by the voltage, membrane type, and the feed-water source. According to the authors, these factors have main impacts on the process eco-efficiency [97], but no EE score was calculated to assess such revendication. One of the main advantages of EDM is the absence of precipitates in the system thanks to the conversion of lightly soluble salts into highly soluble salts. Indeed, EDM is an ED with four compartments of four alternated IEMs with the presence of a solution providing exchangeable ions for the metathesis reaction. For example, from a solution containing magnesium chloride and sodium sulphate, a solution of magnesium sulphate and sodium chloride is produced and desalinated [98]. EDM allowed a less expensive disposal of higher concentrated brackish waters compared to RO. The study would have been enriched by a comparison of the eco-efficiency scores of RO and EDM for desalination.

Single- and multi-batch electrodialysis was also used to recover nitrate from municipal wastewater. The study aimed to optimize and enhance the energy consumption of this process [99]. The more efficient parameters were a flow rate of 60 L.h^−1^, four cell pairs, and a ratio of diluted to concentrated solutions of 4 (2/0.5). The authors were able to recover almost all the nitrate for an energy consumption of 1.44 kWh · kg^−1^. A two-batch stage ED was also tested, which means that the concentrated solution of the first stage became the feed of the second stage. This allowed the production of fresh water from the first batch and a more concentrated product from the second. The energy consumption of the two-batch stage ED was 4.34 kWh · kg^−1^. High recovery efficiency was demonstrated from 460 mg.L^−1^ NO_3_^−^ in the first stage and 1920 mg.L^−1^ NO_3_^−^ in the second stage. The calculation of the energy consumption for the recovery of 1 kg of NO_3_^−^ was based on the volume of the concentrated solution, excluding the electrode compartments, and the energy needed for pumping was not included in the calculation. Moreover, the voltage across the electrode compartments in consideration was not mentioned in the determination of the cell’s voltage [3]. The impacts of the electrode rinsing solution were also demonstrated as an important parameter in nitrate recovery. Na_2_SO_4_ showed the highest recovery efficiency with minimal energy consumption. Finally, a volume of 8 L of water was recovered for 0.5 L of a concentrated stream with low energy consumption. The concentrated nitrate solution from domestic wastewater might then be used as a fertilizer [99]. The optimization of the energy consumption and the concentration of nitrate in the final product are parameters that impact the calculation of an EE score. The authors could have used this method to compare the different processes proposed or compared with conventional methods to support the interest of ED for these applications.

Concerning the seawater desalination field with ED processes, no study has tried to quantify the EE of the electrodialysis process through comparable scores. However, seawater reverse osmosis desalination treatments’ eco-efficiencies were quantified through environmental LCA and life-cycle cost assessment [100,101,102]. These works showed the feasibility of and interest in quantifying EE to demonstrate the best process. This is a missing point in ED studies that could help industries to point out the best option for real applications and highlight the potential of ED.

### 4.2. Food Industry

**Figure 7:**
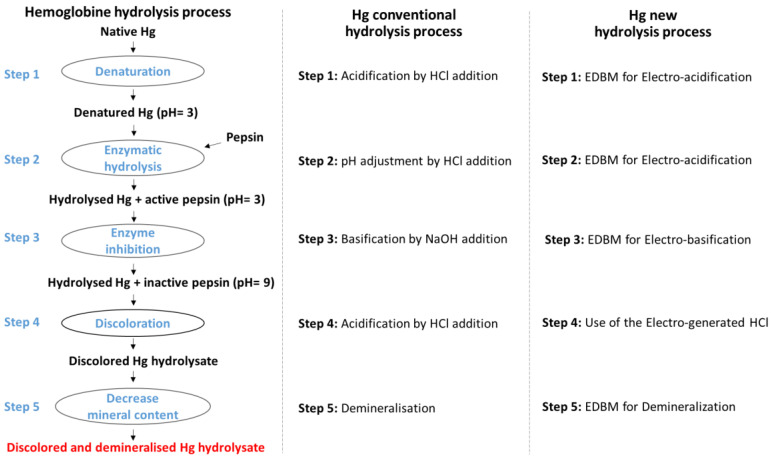
Comparison of the conventional and novel process using EDBM for bovine hemoglobin hydrolysis. EDBM: electrodialysis with bipolar membrane; Hg: hemoglobin.

Effluents or by-products from the food industry can be valorized by enzymatic hydrolysis to produce peptides. A recent study on bovine blood showed the ability to perform all the steps of peptide production simultaneously using EDBM [103,104]. The authors proposed a new sustainable process able to realize the denaturation of protein, hydrolysis, the inactivation of the enzyme, discolouration, and the demineralization of the hydrolysates in a “4-in-1” system. An important difference from the conventional peptide production process is the absence of chemicals (Figure 7) [104]. Indeed, no external chemicals are needed apart from the enzyme. The capacity of EDBM to perform electro-acidification and electro-basification allowed the denaturation of the hemoglobin at pH 3, its enzymatic hydrolysis, which must be achieved at a constant acidic pH; and the enzyme inhibition, which requires a pH value of 9. No HCl nor NaOH were used to adjust the pH, compared to the conventional hydrolysis process. Moreover, the final hydrolysate had a lower mineral content as a result of demineralization occurring in the system [105] due to the special membrane stacking in the EDBM configuration. For applications in food preservation or pharmaceuticals, a low salt content (NaCl having a hypertensive effect) is important to avoid health problems due to the absorption of additional salt by consumers. Moreover, the presence of a high concentration of salts could impact the structure of hemoglobin, the activity of enzymes, the purity of the final product, and the antimicrobial activities [106]. To enhance the commercial potential of the hydrolysate, discolouration can also be required: the dark brownish colour conferred by hemoglobin to food formulations restricts its use as an ingredient in meat products, such as light-coloured deli meat [107] or poultry minced meat. However, the heme, the source of colour and an iron-rich molecule, can then find applications in animal feed or pharmaceuticals. In this “4-in-1” process, electro-generated HCl was used to realize the precipitation of the heme. During this new hydrolysis process, an important mechanism called “zipper”, usually obtained with a conventional method, was maintained [103]. This hydrolysis mechanism is essential to obtain the desired antimicrobial peptide population in the final hydrolysate. The production of antimicrobial and antioxidant peptides was demonstrated [105], but also the presence of opioid, hematopoietic, potentiator of bradykinin, hypolipidemic, bacterial growth stimulator, and antihypertensive known peptides were highlighted [104]. This process of bioactive peptides production, without the addition of solvent, is a new eco-efficient process. However, no EE score was calculated to compare the conventional process and the new process.

### 4.3. Chemical Industry

The authors compared the use of EDBM with electro-ED (EED) to generate sodium hydroxide from spent caustic, which is an important threat to the environment [108]. EED is different from EDBM in terms of water splitting since water dissociation phenomena occur due to reduction (production of OH- from water dissociation) and oxidation (production of H+) reactions, respectively, at the cathode and anode [108] instead of at the membrane interface in EDBM. They showed that EDBM allowed a higher base yield and current efficiency, but at a higher cost compared to EED (respectively, 0.97 $/kg NaOH against 0.86 $/kg NaOH). However, in terms of industrial scale, the economics of EDBM would be lower than EED since in the EDBM stack, only one pair of electrodes is needed with multiple BM to increase the surface of the effective membrane and hence the efficiency of the process while increasing the productivity of EED. The stacking of more electrodes is required, which increases the cost of the process by a lot, as already demonstrated previously by Bazinet et al. [109] for soybean protein production. This process presents more environmental benefits in the long term by preventing secondary pollution [108]. The authors could have performed the calculation of an EE score to support their demonstrations more strongly.

EDBM was proposed to replace the conventional production of salicylic acid, for more eco-efficient and cleaner production [110]. Indeed, salicylic acid production needs a large amount of sulfuric acid and generates large volumes of sodium sulphate and waste [111]. The process was optimized in 2015 using an aqua-ethanol mixed solution in the system to increase the solubility of the salicylic acid. The energy consumption was optimized by the modification of the current density, the concentration of ethanol content, and sodium salicylate. This alternative method proposed is significant and a step toward the cleaner production of water-insoluble aromatic acids [111]. The conventional production and the EDBM could be compared through the calculation of EE scores to support the importance of this method.

EDBM might be used to separate solutions of captured CO_2_, usually under aqueous carbonates or bicarbonates forms, to regenerate pure CO_2_ [112]. The most well-known method to separate CO_2_ is the Carbon Capture and Sequestration (CCS) approach. Nevertheless, CCS has no ability to capture CO_2_ directly from the atmosphere. The direct capture of CO_2_ is necessary and of major interest since one gallon of motor gasoline releases around 19.4 lbs of CO_2_ into the atmosphere [113]. Hence, EDBM was evaluated to propose a more eco-efficient method by regenerating pure CO_2_ from potassium carbonate and bicarbonate solutions. In this EDBM application, the stack of membranes was composed of the alternate stacking of AEMs and BMs. Under the electrical current, CO_2_ transported through CO_3_^2−^ or HCO_3_^−^ ions migrates to the acidic solution, where it is converted into CO_2_ gas. The efficiency was evaluated by the calculation of energy consumption. The total energy cost was about 300 kJ · mol^−1^ CO_2_ for capture and regeneration, and the authors compared it to the energy consumption of liquid fuel synthesis. Indeed, 300 kJ · mol^−1^ CO_2_, in the context of methanol synthesis and considering that one mole of CO_2_ is equivalent to one mole of methanol, corresponds to only 19% of the total energy needed [112]. An alternative EDBM process was also tested: high-pressure EDBM, to have better control of the CO_2_ gas desorption [114]. The energy consumption of EDBM CO_2_ desorption decreased by 29% compared to operation at 1.5 atm (standard). To support these results, a comparison using EE scores between the EDBM and high-pressure EDBM or compared to another method would have made it even stronger.

EDBM was proposed for the production of glycerin from diester wastes [115]. The authors conducted energy consumption and economic evaluations. An important part of the process improvement was demineralization since the final concentration of glycerin is often limited by the presence and precipitation of sodium sulphate [115]. The method allowed the demineralization of 80% of a solution containing 65% glycerin. Moreover, sulfuric acid and sodium hydroxide solutions were produced and recycled in the process. The sodium hydroxide might be used in the demucilation or neutralization steps before obtaining diester waste. The sulfuric acid might be reused as a neutralization solution to obtain the glycerin solution. Following the EDBM step, the glycerin solution produced could be sold at a price 1.4 times higher than the conventional or initial one. Consequently, this study demonstrated the importance of considering the long-term economic benefits in addition to the EDBM cost development. EE score determinations would put into perspective the advantages acquired in terms of environmental and economic impacts.

As can be observed through the different studies presented in this section, ED technologies alone or coupled with other technologies are interesting avenues to obtain the most eco-efficient global processes. However, it appeared here that, when the authors speak about EE, mainly the technological efficiency of the process is studied since the main components described and compared to assess “eco-efficiency” are processing parameters such as energy consumption, production rates, current efficiency, etc. Hence, the conclusions on EE are mainly based on the potential of these technologies, but the environmental impacts induced by the technological changes are not always considered. Hence, there is still a lack of studies using EE scores to support their conclusions when assessing the EE of their process.

## 5. Electrodialysis and Circular Economy

The importance and interest of EE to support decision-making in the context of cleaner production were stated in the first part. In the same context, the circular economy (CE) takes on a growing role. Throughout different industrial fields, CE showed its importance in moving toward cleaner and more sustainable production. First, CE in the desalination of seawater and brine is presented, then in the chemical, metallurgical, and mining industries, and finally in the food industry.

### 5.1. Desalination of Seawater and Brine

Instead of producing one final product, the desalinated water, and a concentrated stream dangerous for the environment, the desalination of seawater can be applied in a circular economy. Indeed, the concentrated streams can be treated and valorized. Conventional ED has demonstrated the production of fresh water and highly concentrated brine (27.13% (*m*/*v*)) [96]. In order to obtain coarse salt from the concentrated brine, the acceptable concentration of the solution for the evaporation method to produce salt is 17% (*m*/*v*) [96]. Edible salt can then be produced from this problematic effluent using ED and further treatment to remove impurities. In parallel, the fresh water generated can be released into the environment to regenerate the natural resource. The wastewater is consequently the raw material of this new process. Another interesting valorization of seawater RO concentrated streams is to use EDBM to produce solvents such as HCl (~3.3 mol·L^−1^) and NaOH (~3.6 mol·L^−1^) [116]. To obtain commercial concentrations of HCl, the authors proposed combining the process with distillation. From seawater RO brines, it is possible to produce solvents that are marketable or usable within the same industries for cleaning or for processes requiring solvents. Instead of being considered waste in a linear economy, this brine enters a new loop. The evaluation of a pilot-scale EDBM treatment plant feasibility and performance for this purpose were investigated [117].

### 5.2. Chemical, Metallurgical, and Mining Industries

Concerning the chemical, metallurgical, and mining industries, one of the main issues is their wastewater. The following part presents different ways of using those wastewaters with electrodialysis technologies to add new values and create a loop to access circular economies.

#### 5.2.1. Recovery of Acid and/or Base

In the chalcopyrite mining industry, a large amount of acidic wastewater is produced during the process. By using a conventional ED system, a demonstration of the capacity to recover 95–98% of the sulfuric acid was performed [118]. A cationic ion-exchange process was used as a pre-treatment process to remove heavy metals before the ED system and produce a reusable sulfuric acid solution. The authors showed that the sulfuric acid concentrate and the water obtained after ED were both of acceptable quality to be reused in the chalcopyrite mining industry [118]. An interesting example was also demonstrated in the aluminum finishing industry [119]. The aim was to use ED to recover acid and water that can be reinjected into different steps of the process, the aluminum anodizing and rinsing steps, respectively. According to the authors, the wastewater could be decreased by 90% and only 10% would still be sent to wastewater treatment plants [119].

In the chemical industry, another type of wastewater is collected: high-salinity or saline wastewater. The sodium sulphate salt from chlorine dioxide generators can be treated by EDBM to produce sodium hydroxide and sulphuric acid [120]. A multistage-batch EDBM for base production was proposed for valorization [3]. This system is based on the adjustment of the acid, base, and salt solution ratios in the compartments of the EDBM to control the concentration gradient, which is often high in an EDBM system converting salt into acid and base. Indeed, the process performance is based on the adjustment of the ratio in the system. The stack was formed of four acid compartments, four base compartments, and four feed compartments. Depending on the number of stages, the number of EDBM stacks varied (a two-stage batch involves two stacks). The two-stage-batch EDBM with a change of the acid solution before the second batch was highlighted as the most effective way [3]. The study demonstrated that this new process increased the base concentration obtained (3.4 mol · L^−1^) and extended the viability of the global process by reducing energy consumption (1.54–1.9 kWh · kg^−1^ ). Indeed, additional studies showed the capacity of EDBM to treat those wastewaters, but the concentration in acid and base solutions was lower, around 2.0 mol · L^−1^ [121,122]. Moreover, a study by Herrero-Gonzalez et al. [123] obtained a concentration of up to 3.6 mol · L^−1^ for the base, but the energy consumption in their system was between 21.8 and 43.5 kWh · kg^−1^. These results support the interest in the multistage-batch EDBM. The fresh water obtained can be safely disposed of or reinjected into the processes as the solvent produced. This allows the establishment of a circular economy. The authors proposed a more productive way to create valuable industrial solvents from waste; however, this method has not yet been performed at the pilot scale.

A similar demonstration was performed on high-salinity sulfanilic acid wastewater. EDBM was used to separate sulfanilic acid from NaCl, which was simultaneously converted into HCl and NaOH [124]. Another interesting study used SED as a pre-treatment for EDBM. The aim was to separate monovalent and divalent ionized salt (Na_2_SO_4_ and NaCl) before the production of a base, sodium hydroxide (NaOH), and acids, hydrochloric acid (HCl) and sulfuric acid (H_2_SO_4_). This additional step of SED allowed the treatment of a solution containing different salts at distinct concentrations [125]. The recovered acids and bases were then proposed to be reinjected into the same process to achieve a circular economy.

Neopentyl glycol (NPG) is an organic compound used in the synthesis of polyesters, paints, plasticizers, etc. Its production generates large amounts of salt wastewater and the consumption of large amounts of acids, bases, and water [126]. EDBM was proposed to reach a sustainable valorization of NPG waste salt. The method showed the capacity to convert NPG waste into high-value formic acid and soda, which are necessary for NPG production. Indeed, they can be valorized as raw materials for upstream hydrogenation and neutralization processes [126].

#### 5.2.2. Recovery of Valuable Compounds

Wastewater containing metal and organic matter has poor biodegradability and high toxicity. A method of complexation by ED to recover acids, molecules, and salt was proposed to treat them [127]. A complexation between heavy metal and neutral organic compounds, before ED, produced positively charged complexes able to migrate in the system. The authors were able to recover 99.4–99.5% of metal ions and 97.8–99.9% of organics. Chromium was recovered in metallo-organic form (Cr(acac)_n_^(3−n)+^) or as oxide nano-powder after post-treatment [127]. Another study showed the feasibility of using EDBM to recover chromium. This method involved the oxidation reaction of Cr^3+^ using H_2_O_2_ and hydroxides produced by the BM. The chromium is then recovered in Na_2_CrO_4_ form [128]. The authors also demonstrated that the increase in the number of recovery chambers allowed an increase in current efficiency and a decrease in specific energy consumption [128]. The chromium recovered then has several opportunities to take part in new processes and create a loop instead of becoming waste. The fields of application are electroplating, metal processing, glassmaking, ceramics, electronic equipment, and more [129].

Pig manure hydrolysates are rich in nitrogen, phosphorus, and organic matter such as volatile fatty acids. EDBM was used to recover NH^4+^ in the basic compartment and PO_4_^3−^ and volatile fatty acids in the acid compartment [130]. The stack of membranes was made of a repeated unit of one BM, one AEM, and a CEM, followed by another BM. The authors proposed the use of a two-stage EDBM to optimize the separation. Indeed, the base generated in the first and second stages was used to recover NH_3_ using air stripping. The first acid was reused in a loop to acidify the raw pig manure. PO_4_^3−^ and volatile fatty acids were recovered in the acid compartment of the second-batch stage. Finally, the remaining base solution can be mixed with the second acid to form struvite, in order to harvest the phosphorus. The aim of this study was to recover all resources and close the loops. It is a great example of ED application in a CE [130].

The importance and feasibility of separating rare earth elements, composed of 15 lanthanides and two similar chemical elements, scandium and yttrium, from wastewater using electrodialysis technology were also reported [131]. Hence, to reduce the primary extraction of mining ores, the main objective of using ED technologies is to explore secondary resources, such as mining effluents or industrial wastewater, to extract rare earth elements. Indeed, these rare earth elements are critical raw materials for many modern technologies, such as electric vehicles, electronic devices, high-performance magnets, and aeronautics [131,132]. To overcome the difficulty of their primary extraction, ED was proposed to be used on streams containing those interesting compounds. The technology was tested to separate and concentrate scandium (Sc) ions in the Sc^3+^ form. The authors managed to perform a ~99.5% removal [131]. Hence, recovering rare earth elements from modern technology industries’ wastewater and being able to recycle them in their own production lines, after applying ED, allows the formation of the necessary loop in the context of a circular economy.

The ED process coupled with other separation methods was also shown as pertinent. Indeed, a methodology of integrated nanofiltration–diafiltration with an electrodialysis process for the sustainable extraction of dyes, NaCl, and pure water from high-salinity textile wastewater was studied [133]. The authors extracted useful resources that can be reused. Indeed, the salts were separated from the dyes with high purity and then NaCl was concentrated with ED to produce pure water. The final salt generated might be used for commercial purposes and the pure water could be used to provide the nanofiltration–diafiltration unit of the first step [133] to close the loop.

Finally, in the nickel electroplating industry, an industrial scale-up of an ED-based recovery process from wastewater was tested. The ED system was directly connected to the rinse-water line of the nickel plating. A concentrated stream of nickel and water was recovered after ED treatment. By creating this loop, this improved processing line allowed chemical savings of 20% for the nickel plating baths and reduced the volume of wastewater by 90% [134]. Indeed, the nickel and water recovered had a sufficient quality to not negatively impact the final product (visual quality and protective properties of the deposit). They were used for reinforcement in the nickel batch or as rinsing water in the process [134]. It is considerable and demonstrates the real application possibilities in the industry.

### 5.3. Food Industry

Another industry known to produce a high volume of effluents, wastewaters, and liquid by-products is the food industry. Several paths to valorize these products in a circular economy are presented.

#### 5.3.1. Recovery of Acid and Base

During cheese or yogurt manufacturing, large volumes of whey (salty, acid or sweet) are generated. In the world, about 24 million tons of cheese are produced which generates approximately 21.6 million tons of cheese whey [135]. A study suggested the use of EDBM to transform the salty whey into chemicals: NaOH and HCl [10]. The aim is to further use them for clean-in-place and ion exchange resin regeneration in the factory. The authors demonstrated the importance of the pre-treatment of whey, especially the precipitation of calcium phosphate with base. The interesting part is the possible use of the produced base for this pre-treatment as a loop [10]. It is important to mention that the recovered salty whey is generally also acidic due to the significant presence of another interesting compound: lactic acid. Lactic acid is a really valuable organic acid with applications in different industries and is also one of the main limitations for whey valorization in a powder form. Indeed, the presence of high levels of lactic acid and calcium decreases the glass transition of lactose, which generates an undesirable sticky powder after drying [136,137]. Different combinations of membrane processes, ultrafiltration, nanofiltration or dia-nanofiltration, and electrodialysis showed encouraging results to recover lactic acid on one hand and recover ideal whey for drying on the other hand. Hence, from one co-product, new products were produced and usable [138,139,140]. Solvents (NaOH and HCl) were reinjected in the cheese production line: lactic acid used as a preservative solution in the dairy industry and dryable whey to be commercialized. This allowed for closing the loop and valorizing all by-products.

During the production of wine, the feasibility of integrating ED and EDBM to recover tartaric acid was demonstrated. Distilled vinasses were treated first with ED to concentrate the potassium hydrogen tartrate (KHT). This concentrate was then treated by EDBM and, due to the particularities of bipolar membranes, tartaric acid (H_2_T) and potassium hydroxide (KOH) were produced [141]. Several other studies confirmed the adaptability of EDBM to recover organic acids, which were also purified from fermented waste and a diversity of wastewaters. A demonstration of the feasibility was also carried out on a model solution from beet molasses feed used to produce sugar. EDBM was applied, combined with monovalent anion-selective membranes [142]. Once again, valuable products were generated from by-products.

#### 5.3.2. Recovery of Protein Compounds

Other types of valuable molecules found in waste or by-products are proteins or protein-derived compounds.

Concerning proteins, valuable proteins are found in dairy effluents, and it was demonstrated that ED with a filtration membrane (EDFM) is able to separate them from other proteins. Hence, lactoferrin and immunoglobulins were isolated from dairy whey. They were retained in the feed compartment while the rest of the dairy protein went through the membrane [85,143]. Migration up to 8.9 g/m^2^ · h for lactoferrin [85], 41.0 g/m^2^ · h for β-lg [143], and 29.1 g/m^2^ · h for BSA [85] in model whey or whey-enriched solutions were reported. Very recently, EDFM was used for growth-factor recovery [144].

The valorization of protein may also be carried out by the production of bioactive peptides. However, the production, generally using enzymatic hydrolysis, is not always enough to generate a global hydrolysate with bioactivities. The fractionation step is of importance to highlight the bioactivit(y/ies) by the concentration/separation of specific peptides. Indeed, the hydrolysis of high-molecular-mass proteins produces a large population and variety of peptides, which are not all bioactive and dilute the bioactivity of the peptides of interest. Hence, the capacity of EDUF to separate positively (basic) and negatively (acid) charged peptides individually or simultaneously from β-lactoglobulin hydrolysate was demonstrated in the pioneering works of Poulin et al. [78]. The goal was to purify specific peptides such as ALPHMIR, a well-known ACE-inhibitory peptide to enhance the final antihypertensive bioactivity of the fraction, which can find applications in biopharmaceuticals [78]. This ACE-inhibitory peptide, β-lg 142–148, presented the highest migration rate with a value of 10.75% [78]. This study is part of the numerous works on the pertinence of EDUF to give a second life to valuable compounds. Moreover, this process was tested very recently and for the first time at a semi-industrial scale [39,40,145]. The potential to valorize β-lactoglobulin was also investigated deeper with the demonstration of an electrodialytic reactor, based on an EDUF configuration, for the enzymatic hydrolysis of the protein and the simultaneous fractionation of generated bioactive peptides in one step. Bioactivities such as hypocholesterolemia, antihypertensive, and antibacterial activities were found for the recovery fraction [83].

**Figure 8:**
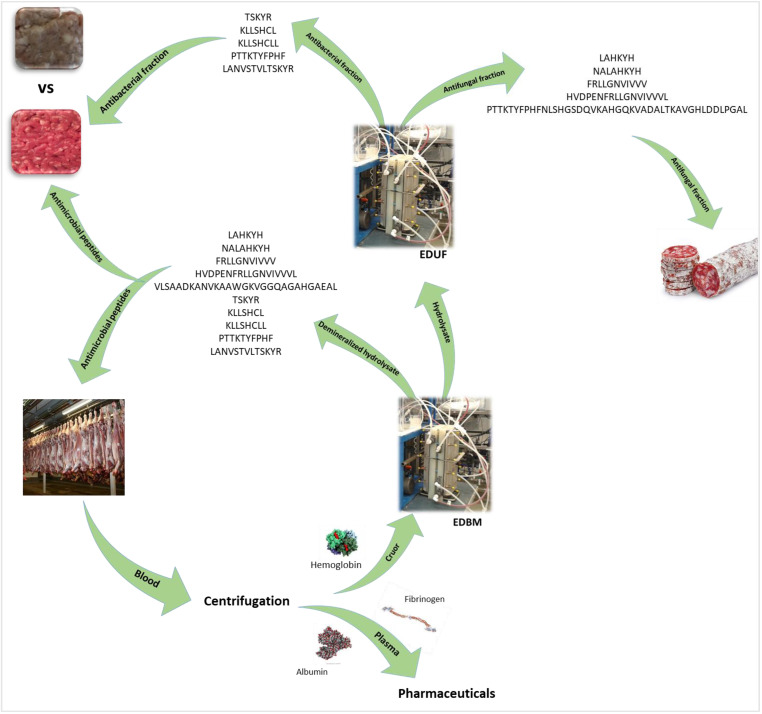
Diagram of the valorization of blood, coproduct from slaughterhouses, using ED technologies, toward a circular economy. EDBM: electrodialysis with bipolar membrane; EDUF: electrodialysis with ultrafiltration membrane. Peptides are represented by their amino acid sequences, e.g., LAHKYH.

In the Canadian meat industry, an important volume of blood, with a high concentration of protein, is produced: 55 and 73 million litres per year for cattle and pigs, respectively [146]. These volumes are recoverable and represent valuable sources of proteins. The production of bioactive peptides from protein contained in wastes or by-products is a promising valorization path toward a circular economy. The final hydrolysate was demonstrated to be an alternative to chemical additives such as butylated hydroxyanisole (BHA) or butylated hydroxytoluene (BHT) used for meat preservation in terms of microbial growth and lipid oxidation [147] and suspected to induce pathological and toxic effects [148,149]. This hydrolysate can be used as a natural and safe preservative of meat products (Figure 8). So, the blood becomes the raw material of enzymatic hydrolysis by EDBM, in the framework of a circular economy, and the new product is used in the meat industry to close the loop. It is also possible to go further by opening a new loop using the hydrolysate produced by EDBM and separating its different peptides by EDUF (Figure 8). Indeed, EDUF was used on bovine hemoglobin hydrolysates and showed interesting results in fractionating the hydrolysate and improving its bioactivities [150,151,152]. Moreover, in recent studies on porcine and bovine hemoglobin molecules [105,153], as well as porcine cruor [154], antifungal activities were demonstrated. Specific antifungal peptides were discovered in the porcine cruor study. The EDUF process could be used to fractionate antibacterial peptides such as neokyotorphin (TSKYR) (Figure 8) and concentrate the feed fraction in antifungal ones (FRLLGNVIVVV, LAHKYH, NALAHKYH, etc.). This could lead to the production of two fractions both useful for different meat products, such as fresh meat or ham for antibacterial peptides, and deli meats such as sausage and saucissons for antifungals. Consequently, from blood, two fractions with different bioactivities and with different applications for meat products are generated and avoid new by-products. The loop is then obvious and highlights once again the potential of blood from slaughterhouses to enter a circular economy (Figure 8).

Other protein-based by-products showed the capacity to be valorized with EDUF: amongst others, alfalfa white protein hydrolysate [79], snow crab by-product hydrolysate [77], or salmon protein hydrolysate [155]. Various bioactivities were obtained, after EDUF separation, with potential applications in pharmacology, nutrition, food preservation, etc.

## 6. Electrodialysis as an Eco-Efficient Process in a Circular Economy

Some studies have gone deeper into sustainability by investigating the improvement of the eco-efficiency of processes simultaneously to a transition from a linear to a circular economy by creating loops.

### 6.1. Cranberry Industry

**Figure 9:**
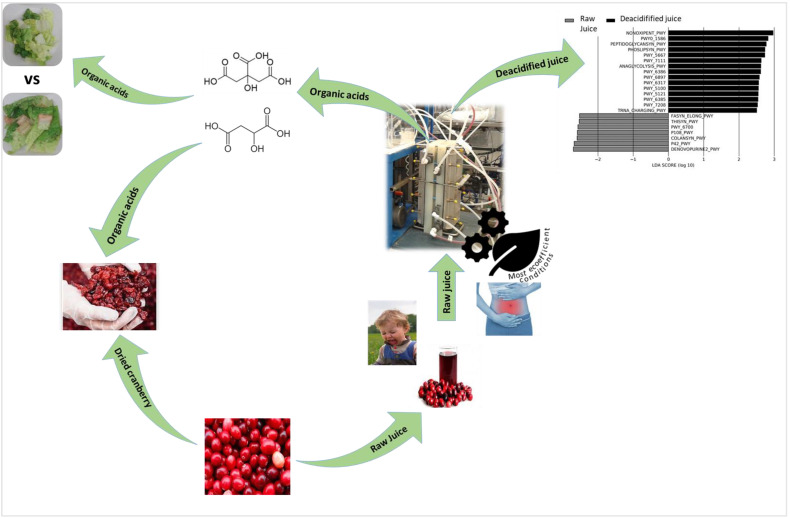
Diagram of eco-efficiency improvement in the cranberry industry, in the context of a circular economy.

Through different studies conducted on the applications of ED in the cranberry transformation field, a final production line combining EDBM as the most eco-efficient process in a circular economy was proposed (Figure 9), since the two main products from cranberry transformation are dried cranberries and juice.

The deacidification of cranberry juice is a necessary step to increase its consumption by the population. Indeed, it contains a high concentration of citric, malic, and quinic acids, causing an undesirable taste [156,157] and health side effects [158]. It has also been demonstrated in vitro that citric acid in cranberry juice was the main organic acid responsible for the disruption of intestinal Caco-2 cells and thus the barrier integrity [159,160]. Different deacidification methods already exist for wine and fruit juices, such as ion-exchange resins and salt precipitation. However, recently, EDBM was demonstrated to slightly increase the pH of cranberry juice but to decrease drastically and selectively the content of organic acids [161]. Such a selective migration of organic acids using EDBM had never been reported previously in the literature. These results were even more important, as preserving quinic acid in the treated juice is essential. Indeed, this acid is used for cranberry juice authentication [162,163].

In order to increase the value of cranberry juice while limiting the environmental impacts, some studies calculated EE scores to support which process would be the best choice. First, EDBM was compared to the ion-exchange resin process [32]. The scores were calculated taking into account the functional value of the food product, an approach closer to consumers’ interests [32]. Amongst others, the final concentration of polyphenols, molecules recognized as having health benefits, in the deacidified juice was taken into account when calculating EE scores (Table 1). Considering the removal of harmful acids, ion-exchange resins were more eco-efficient than non-deacidified juice, but regarding polyphenol content, IE resins were less eco-efficient. In addition, EDBM was able to extract organic acids in functional forms without effluents such as the ones needed to regenerate the ion-exchange resin (four volumes of concentrated base for each volume of juice treated). If they are used in dried cranberry production, EDBM is the more eco-efficient process [32]. EDBM was also compared to another deacidification method: salt precipitation [33]. The environmental impact evaluation of both processes was based on LCA. EE scores demonstrated that EDBM was the most eco-efficient method, with up to 20.6% more EE compared to precipitation by salts [33,164]. Another way of increasing the EE of the process was the reuse of the organic acid recovery solution to increase its concentration for further uses and to reduce energy consumption by up to 42.9% with three reuses [164]. Concerning the deacidified juice by EDBM, beneficial effects on gut microbiota were also demonstrated, specifically in the Lachnospiraceae family. These microorganisms are linked to the effects and functions associated with the protection of the intestine [156]. Following all these demonstrations, the EDBM deacidified juice, with a better taste and improved functionality, can be marketed, while the organic acids fraction, the coproduct, can be valorized. Indeed, organic acids are used for the adjustment of organic acid content in dried cranberries and as food preservative agents in other products, such as lettuce, to protect against microbial proliferation [165] and browning [166,167]. The loop is then closed (Figure 9) and shows how eco-efficient ED processes might be applied in a circular economy in the cranberry industry.

### 6.2. Dairy Industry

**Figure 10:**
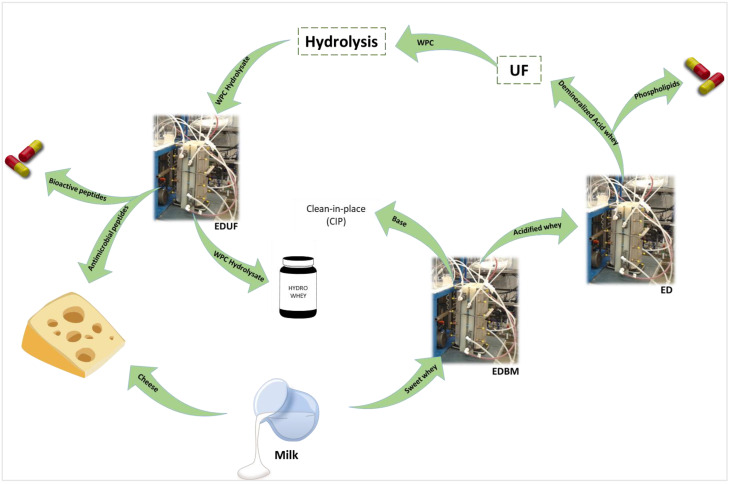
Diagram of different ways to improve the eco-efficiency of the dairy industry in the context of a circular economy. ED: electrodialysis; EDBM: electrodialysis with bipolar membrane; EDUF: electrodialysis with ultrafiltration membrane; UF: ultrafiltration; WPC: whey protein concentrate.

Eco-efficiency in the context of a circular economy is also reachable in this industry thanks to ED technologies (Figure 10). ED technologies or a combination of ED technologies can be used to valorize different molecules (whey proteins, phospholipids, lactic acid, minerals, etc.) from whey, which is the main by-product.

Whey proteins, such as β–lactoglobulin and α–lactalbumin present in the whey, might be valorized through enzymatic hydrolysis to produce peptides and then fractionated by EDUF. Hence, in the context of the industrial application of this technology, two EDUF configurations were compared at a semi-industrial scale-up and adapted to reach the most efficient and selective process. Indeed, CEM/UF/CEM and CEM/UF/AEM configurations of EDUF were tested [39]. They allowed the recovery of 10 predominantly cationic peptides from a tryptic whey protein hydrolysate. Among them, a peptide inhibiting the angiotensin-converting enzyme (ACE), also called an antihypertensive peptide, was found: ALPMHIR [168]. Two antidiabetic peptides, IPAVFK and LIVTQTMK [169], were also identified. EE scores were determined to compare these configurations. The new configuration (CEM/UF/AEM) aimed to diminish demineralization and unbalanced conductivity. Finally, it allowed for decreasing the environmental impact cost by 26.5% and increased bioactive cationic peptides recovery by 18% [39].

In the cheese field, sweet whey and some derived products, such as whey protein concentrate (WPC), are produced in large volumes, and both contain residual lipids (3–7%), such as phospholipids [170]. Indeed, residual lipids may be still present in sweet whey after the skimming process and consequently in WPC after its concentration by ultrafiltration, as they are not compatible with centrifugation due to the presence of a negative charge that stabilizes them by electrostatic interactions in whey [171,172]. In addition, phospholipid (PL) recovery is pertinent since they present known health benefits and have negative impacts on final dairy products. Indeed, amongst the phospholipids (phosphatidylethanolamine (PE), phosphatidylinositol (PI), phosphatidylcholine (PC), phosphatidylserine (PS), and sphingomyelin (SM)) present in whey, SM and PS are suggested to have a positive impact on neurodegenerative diseases and brain aging as well as on the prevention of colon cancer formation [173,174]. Furthermore, these residual lipids may cause turbidity issues and are susceptible to chemical reactions such as oxidation, generating off-flavors in the final dry WPC or whey powder [170,175].

Methods have been developed to concentrate PLs from several dairy products and by-products, including solvent extraction, chitosan precipitation, thermocalcic precipitation, supercritical fluid extraction, and ultrafiltration (alone or in combination) [171,175,176,177,178,179]. However, all these methods present disadvantages, such as the use of components with unclear legislation, whether their use is more or less appropriate in the food industry, the difficulty of setting up the best parameters for the process at a large scale, the impact on the environment, and effects on the other components of sweet whey and WPC.

In this scheme, sweet whey/WPC was valorized through first the application of EDBM to produce a base and an acidified whey/WPC. The base generated during EDBM could be reused as a cleaning chemical [10], while the acidified whey/WPC, after EDBM and combined with a dilution factor, allows the recovery of these dairy phospholipids [42]. Phosphatidylethanolamine and phosphatidylserine were the main phospholipids concentrated from the initial products after EDBM and a dilution treatment. EE scores were determined to define the most relevant method. Different scenarios were tested to calculate the score, using the EE score equation (Equation (1)). The environmental impact was based on the main issues of the process: water and effluents (Table 1). The value of the product was defined by three different approaches depending on the phospholipid’s concentration calculation. Finally, after comparing the scores and scenarios, the sweet whey (not concentrated in WPC) combined with a 2X or 4X dilution after EDBM seemed advantageous since these conditions had the highest scores [42] and the highest purity in terms of phospholipids. The process was optimized by coupling EDBM with ED instead of diluting the product after the EDBM step. This combination was demonstrated as the most eco-efficient method to recover phospholipids in comparison with dilution and diafiltration after EDBM [42]. Another advantage was that ED applied on acidified whey allows, on one hand, the production of a demineralized acidic whey, and on the other hand, the separation and purification of phospholipids. Hence, as a further step, an ultrafiltration process can be applied to produce WPC from the demineralized acid whey. Such a WPC can be used as an ingredient to produce dairy products or other food products. A further step of valorization was proposed by using EDUF to separate bioactive peptides from the WPC hydrolysate, which can be valorized as preservatives. Indeed, this hydrolysate may contain antimicrobial peptides or pharmaceuticals due to biologically active peptides, according to the enzyme or hydrolysis duration used [39,145]. WPC hydrolysate remaining from EDUF could be dried and valorized through human diets or sportive nutrition. In this global scheme, ED technologies allowed the formation of loops and the valorization of by-products in the dairy industry, specifically from cheese production.

Another coproduct from dairy product transformation is acid whey. The drying of this co-product is mainly limited by the presence of lactic acid and minerals [136,137]. ED was used in previous studies to demineralize and deacidify acid whey using conventional ED and EDBM [180,181] with the application of a continuous current. However, no EE score was calculated, and one of the downsides reported was calcium scaling in the systems, which may greatly impact the eco-efficiency of the process. A pulsed electric field (PEF), a non-stationary regime applying a hashed current or voltage, was used to decrease this scaling during acid whey treatment. When PEFs are applied, the ED system is under the influence of a constant current/voltage (pulse lapse) for a defined duration, and then the current/voltage is turned off for a fixed time (pause lapse). So, during ED with PEF, consecutive pulse and pause lapses of constant durations are applied throughout the treatment. The application of a 25 s/25 s pulse and pause combination demonstrated a decrease in scaling, as well as decreases in system electrical resistance of 32% and energy consumption of 33%. The lactic acid recovery was also increased compared to the continuous current mode by 16% [138,182]; however, no EE scores were reported to assess the eco-efficiency of ED with PEF treatment, whatever pulse/pause conditions were used.

### 6.3. Water Treatment Plant

**Figure 11:**
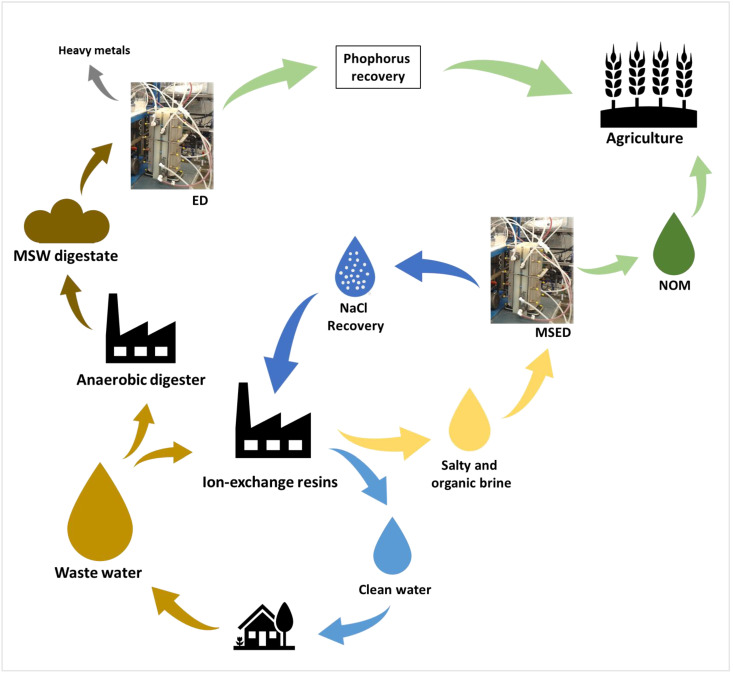
Diagram of the improved eco-efficiency of a water treatment plant using ED technologies in a circular economy. ED: electrodialysis; MSED: monovalent selective ED; MSW: municipal solid waste; NOM: natural organic matter.

Several studies demonstrated the feasibility of using ED technologies to enhance eco-efficiency and recover valuable initially lost components in water treatment plants. Among them, authors have investigated the treatment of ion exchange resin brine to recover salts (NaCl) and organic matter (Figure 11). Indeed, the ion exchange resins step is major to remove these contaminants from water for aesthetic and operational purposes and also to avoid repercussions for human health [183]. Nevertheless, the brine produced is complex to discharge and is polluting. The first solution proposed was the use of ED with conventional membrane or monovalent ion permselective membranes, coupled with a direct current or pulsed electric field (PEF) [184]. The choice of the membrane perm-selectivity and the type of electrical current applied had consequences on the efficiency of the process, especially on membrane fouling [184]. The use of monovalent ion permselective membranes avoids the formation of fouling and allows the production of an NaCl solution of high purity. Another solution tested was to integrate monovalent selective electrodialysis (MSED) and a direct contact membrane distillation system to improve the process’s eco-efficiency [183]. The pure NaCl produced was used to regenerate the resins, which decreased the use of fresh NaCl. Indeed, the solution produced allowed the recovery of up to 85% of the IEC. Thus, brine was seen as an unconventional source of water, minerals, and nutrients [183].

Another possible step in water treatment, anaerobic digestion, also demonstrated interest in using ED technology. The aim was to extract phosphorus from municipal solid waste (MSW) digestate, obtained after the anaerobic digestion (Figure 11). Indeed, lately, phosphate rock must be mined to produce phosphorus for agricultural applications such as fertilizers. The extraction of phosphorus from waste is a sustainable and promising alternative. ED was used to separate dissolved phosphorus anions toward the recovery compartment. During ED, the anions migrate, and the waste solution is not saturated anymore, which allows the dissolution of further phosphorus ions. The concentrated solutions are then used as a fertilizer for agriculture [185,186,187]. Modifications of the ED process were tested to improve the recovery of phosphorus and improve the EE [185]. Consequently, wastewater from water treatment plants has the potential to enter a circular economy using ED technologies as eco-efficient technologies (Figure 11).

## 7. Conclusions

Among different industrial sectors, a significant number of opportunities involving several types of ED technologies to reach EE or CE appeared and were highlighted in the present review (Table 2). It appeared first that ED systems are of major concern or importance in the valorization of water, effluents, and by-products. However, they are still no ideal processes, and improvements can be made. Hence, research on scale-ups and real industrial conditions must be further investigated. Secondly, a lack of demonstration concerning the EE score was reported through this review, although a standardized method exists. Indeed, only a few LCAs were carried out to confirm the EE of proposed ED processes. Such a lack of LCAs was mainly due to the fact that the eco-efficiency analysis, according to the ISO standard and the LCA to be carried out, is a tedious task to undertake. So, to promote and universalize EE scores in many fields, a modification of the methods to calculate standardized EE indicators could be considered. Standardized methods are important to demonstrate the validity of process choices, such as ED, and to facilitate their comparison with other processes.

There are still other ways to go even deeper into the sustainability of processes involving ED. Indeed, recycled materials from discarded reverse osmosis modules were used to produce an ED system. The capacity to produce a system composed of 54% recycled components was demonstrated. They could also be used as membrane support for the preparation of anion exchange membranes [190,191]. The recycling of ED systems and membranes was also investigated [11]. Another way to increase EE and consequently sustainability is to favour the use of renewable energy sources to operate ED [54].

**Figure 12:**
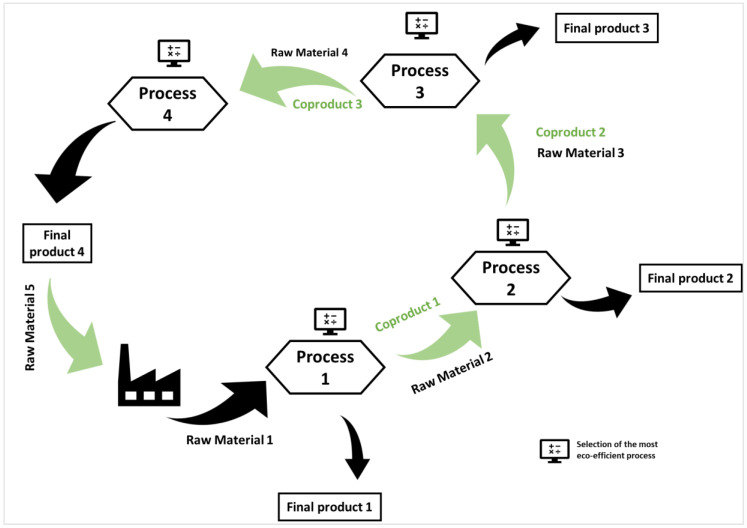
General diagram of the theoretical concept of an eco-circular economy, for a conceptual number of processing steps involved.

More recent studies revealed a new concept that needs to be expanded: the improvement of process eco-efficiency in the context of a circular economy, which could also be named an eco-circular economy. Indeed, a larger number of studies on water, food, mining, and chemistry applying ED were only focused on the concept of a circular economy. This leads to questioning the concept of the accuracy and the EE of the processes included in it. In this context, the idea is not only to make a transition toward a circular economy but to propose the most eco-efficient and circular system: The concept of circular economy is extended to the concept of an eco-circular economy. An eco-circular economy is an optimal scenario between eco-efficient processes and loops, avoiding the production of wastes and allowing the valorization of all streams (Figure 12). Depending on the application fields and the products, the number of process steps and sub-loops should vary. The ideal scenario would be to choose processes with the ideal EE score and continuously improve them by updating them with new parameters, technologies, or energy. ED technologies have shown interesting achievements in eco-efficiency; hence, the interest in investigating them in order to attempt the development of an eco-circular economy is significant.

## Figures and Tables

**Table 2:** Summary of ED applications concerning eco-efficiency, circular economy, and improved eco-efficiency in a circular economy, in different fields.

Concept	Field of Application		Applied ED Process	Reference
Eco-efficiency	Water	Seawater desalinization	Reverse ED on RO brine	[94]
			Conventional ED on RO brine	[96]
			ED metathesis on seawater	[97]
		Water treatment plant	Single- and multi-batch ED	[99]
	Food industry	Meat industry	EDBM	[103,105,106]
	Chemical industry	General	EDBM/EED	[108]
		Salicylic acid	EDBM	[110,111]
		CO_2_	EDBM	[112,114]
		Glycerin	EDBM	[115]
Circular economy	Water	Seawater desalinization	Conventional ED EDBM	[96] [116]
	Mining industry	Chalcopyrite	Conventional ED	[118]
		General	Multistage-batch EDBM	[3]
	Chemical industry	General	EDBM SED Complexation by ED	[117] [125] [127]
		Sulfanilic acid	EDBM	[124]
		Nickel electroplating industry	Conventional ED (Industrial scale)	[134]
		Aluminum finishing industry	Conventional ED	[119]
		Neopentyl Glycol	EDBM	[126]
		Separation of rare earth elements	Conventional ED	[131]
	Textile industry	General	Conventional ED	[133]
	Food industry	Dairy industry	EDBM ED with PEF ED EDFM EDUF	[10] [138] [139,140] [85,143,144] [78,83]
		Meat industry	EDUF + EDBM	[105,150,151,152]
		Seafood processing	EDUF	[77,155]
		Wine industry	ED + EDBM	[141]
		Fermented broth	EDBM with monovalent selective AEM	[142]
Combined concepts	Food industry	Cranberry industry	EDBM	[32,33,156,164]
		Dairy industry	ED EDBM EDUF	[10,39,42,145,180,181,182]
	Water	Water treatment plant	MSED ED (dual-stage extraction)	[184,185,186,187,188,189]

## Abbreviations

AEM	Anion-exchange membrane
CCS	Carbon Capture and Sequestration
CE	Circular economy
CEM	Cation-exchange membrane
ED	Electrodialysis
EE	Eco-efficiency
EED	Electro-electrodialysis
EDBM	Electrodialysis with bipolar membrane
EDFM	Electrodialysis with filtration membrane
EDM	Electrodialysis metathesis
EDNF	Electrodialysis with nanofiltration membrane
EDUF	Electrodialysis with ultrafiltration membrane
FM	Filtration membrane
IEC	Ion-exchange capacity
IEM	Ion-exchange membrane
LCA	Life-cycle assessment
MSED	Monovalent selective electrodialysis
MWCO	Molecular weight cut-off
NPG	Neopentyl glycol
OECD	Organisation for Economic Co-operation and Development
RED	Reverse electrodialysis
RO	Reverse osmosis
SED	Selectrodialysis
WPH	Whey protein hydrolysate
WPC	Whey protein concentrate

## References

[1] United Nations United Nations Conference on the Human Environment Stockholm 1972. https://www.un.org/en/conferences/environment/stockholm1972.

[2] Noronha L. Why Does Stockholm+50 Matter? What Did It Achieve? What Does It Offer Going Forward?. http://www.stockholm50.global/news-and-stories/why-does-stockholm50-matter-what-did-it-achieve-what-does-it-offer-going-forward.

[3] Hussain A., Yan H., Ul Afsar N., Jiang C., Wang Y., Xu T. (2022). Multistage-Batch Bipolar Membrane Electrodialysis for Base Production from High-Salinity Wastewater. Front. Chem. Sci. Eng..

[4] Lefebvre O., Moletta R. (2006). Treatment of Organic Pollution in Industrial Saline Wastewater: A Literature Review. Water Res..

[5] Statistics Canada Water Discharge in Mineral Extraction and Thermal-Electric Power Generation Industries, by Type of Final Treatment and Region. https://www150.statcan.gc.ca/t1/tbl1/en/tv.action?pid=3810008101.

[6] Agriculture and Agri-Food Canada Hogs/Pork. https://agriculture.canada.ca/en/agriculture-and-agri-food-canada/canadas-agriculture-sectors/animal-industry/red-meat-and-livestock-market-information/hogs-pork.

[7] Llop A., Pocurull E., Borrull F. (2009). Evaluation of the Removal of Pollutants from Petrochemical Wastewater Using A Membrane Bioreactor Treatment Plant. Water. Air. Soil Pollut..

[8] Minière M., Boutin O., Soric A. (2019). Combination of Chemical and Biological Processes to Enhance the Treatment of Hardly Biodegradable Matter in Industrial Wastewater: Selection Parameters and Performances. Can. J. Chem. Eng..

[9] Adin A., Asano T. (1998). The Role of Physical-Chemical Treatment in Wastewater Reclamation and Reuse. Water Sci. Technol..

[10] Chen X., Chen G.Q., Wang Q., Xu T., Kentish S.E. (2020). Transforming Salty Whey into Cleaning Chemicals Using Electrodialysis with Bipolar Membranes. Desalination.

[11] Bazinet L., Geoffroy T.R. (2020). Electrodialytic Processes: Market Overview, Membrane Phenomena, Recent Developments and Sustainable Strategies. Membranes.

[12] Mensah J. (2019). Sustainable Development: Meaning, History, Principles, Pillars, and Implications for Human Action: Literature Review. Cogent Soc. Sci..

[13] Wanamaker C. (2018). The Environmental, Economic and Social Components of Sustainability: The Three Spheres of Sustainability.

[14] Gray R. (2010). Is Accounting for Sustainability Actually Accounting for Sustainability…and How Would We Know? An Exploration of Narratives of Organisations and the Planet. Account. Organ. Soc..

[15] Keeble B.R. (1988). The Brundtland report: Our Common Future. Report of the World Commission on Environment and Development. Medicine and War.

[16] Caiado R.G.G., de Freitas Dias R., Mattos L.V., Quelhas O.L.G., Leal Filho W. (2017). Towards Sustainable Development through the Perspective of Eco-Efficiency—A Systematic Literature Review. J. Clean. Prod..

[17] OECD (1998). OECD Eco-Efficiency.

[18] Alves J.L.S., Dumke de Medeiros D. (2015). Eco-Efficiency in Micro-Enterprises and Small Firms: A Case Study in the Automotive Services Sector. J. Clean. Prod..

[19] OECD (2010). OECD Eco-Innovation in Industry: Enabling Green Growth.

[20] Tyl B. Eco-Efficience Industrielle Atteindre l’éco-Efficience à Travers l’éco-Conception et l’écologie Industrielle. https://docplayer.fr/38909760-Emar-eco-efficience-industrielle-atteindre-l-eco-efficience-a-travers-l-eco-conception-et-l-ecologie-industrielle-guide-pratique-n-5.html.

[21] Pouliot Y., Doyen A., Bazinet L., Mikhaylin S., Benoit S., Margni M. (2018). Écoefficience des Procédés de Transformation du Lait: Principes, Méthodologies et Applications. Science et technologie du lait. 3e édition (éditeur Jean-Christophe Vuillemard).

[22] ISO ISO 14045:2012. https://www.iso.org/cms/render/live/en/sites/isoorg/contents/data/standard/04/32/43262.html.

[23] Charmondusit K., Phatarachaisakul S., Prasertpong P. (2014). The Quantitative Eco-Efficiency Measurement for Small and Medium Enterprise: A Case Study of Wooden Toy Industry. Clean Technol. Environ. Policy.

[24] Ho T.Q., Hoang V.-N., Wilson C., Nguyen T.-T. (2018). Eco-Efficiency Analysis of Sustainability-Certified Coffee Production in Vietnam. J. Clean. Prod..

[25] Forleo M.B., Palmieri N., Suardi A., Coaloa D., Pari L. (2018). The Eco-Efficiency of Rapeseed and Sunflower Cultivation in Italy. Joining Environmental and Economic Assessment. J. Clean. Prod..

[26] Keating B.A., Carberry P.S., Bindraban P.S., Asseng S., Meinke H., Dixon J. (2010). Eco-Efficient Agriculture: Concepts, Challenges, and Opportunities. Crop Sci..

[27] Masuda K. (2016). Measuring Eco-Efficiency of Wheat Production in Japan: A Combined Application of Life Cycle Assessment and Data Envelopment Analysis. J. Clean. Prod..

[28] Müller C., Elliott J., Chryssanthacopoulos J., Deryng D., Folberth C., Pugh T.A.M., Schmid E. (2015). Implications of Climate Mitigation for Future Agricultural Production. Environ. Res. Lett..

[29] Laso J., García-Herrero I., Margallo M., Vázquez-Rowe I., Fullana P., Bala A., Gazulla C., Irabien Á., Aldaco R. (2018). Finding an Economic and Environmental Balance in Value Chains Based on Circular Economy Thinking: An Eco-Efficiency Methodology Applied to the Fish Canning Industry. Resour. Conserv. Recycl..

[30] Ullah A., Perret S.R., Gheewala S.H., Soni P. (2016). Eco-Efficiency of Cotton-Cropping Systems in Pakistan: An Integrated Approach of Life Cycle Assessment and Data Envelopment Analysis. J. Clean. Prod..

[31] ISO AS ISO 14044:2019 Environmental Management - Life Cycle Assessment—Requirements and Guidelines. https://infostore.saiglobal.com/en-us/standards/as-iso-14044-2019-1156937_saig_as_as_2747947/?gclid=Cj0KCQiA99ybBhD9ARIsALvZavXquoH5Y4RpHHGoOLvhphptFQx3mUofpiLRBsdYvq9XATreYf3CMi8aAov6EALw_wcB&gclsrc=aw.ds.

[32] Chaudron C., Faucher M., Bazinet L., Margni M. (2019). The Cost Is Not Enough—An Alternative Eco-Efficiency Approach Applied to Cranberry de-Acidification. J. Clean. Prod..

[33] Faucher M., Henaux L., Chaudron C., Mikhaylin S., Margni M., Bazinet L. (2020). Electromembrane Approach to Substantially Improve the Ecoefficiency of Deacidified Cranberry Juice Production: Physicochemical Properties, Life Cycle Assessment and Ecoefficiency Score. J. Food Eng..

[34] Houssard C., Revéret J.-P., Maxime D., Pouliot Y., Margni M. (2022). Measuring Shared Value Creation with Eco-Efficiency: Development of a Multidimensional Value Framework for the Dairy Industry. J. Clean. Prod..

[35] Faucher M. (2022). Valorisation écoefficiente du lactosérum doux par procédés électrodialytiques pour la production de fractions phospholipide et peptidique potentiellement bioactives. Ph.D. Thesis.

[36] Jolliet O., Saadé M., Crettaz P. (2010). Analyse du Cycle de vie: Comprendre et Réaliser un écobilan.

[37] Muralikrishna I.V., Manickam V., Muralikrishna I.V., Manickam V. (2017). Chapter Five—*Life Cycle Assessment*. Environmental Management.

[38] Golsteijn L. Life Cycle Assessment (LCA) Explained. https://pre-sustainability.com/articles/life-cycle-assessment-lca-basics/.

[39] Geoffroy T.R., Bernier M.E., Thibodeau J., Francezon N., Beaulieu L., Mikhaylin S., Langevin M.E., Lutin F., Bazinet L. (2022). Semi-Industrial Scale-up of EDUF Technology for the Electroseparation of Bioactive Cationic Peptides: Impact of Process Parameters and Cell Configurations on Eco-Efficiency. J. Membr. Sci..

[40] Geoffroy T.R., Thibodeau J., Faucher M., Langevin M.E., Lutin F., Bazinet L. (2022). Relationship between Feed Concentration and Bioactive Cationic Peptide Recovery: Impact on Ecoefficiency of EDUF at Semi-Industrial Scale. Sep. Purif. Technol..

[41] Agoua R.-S., Bazinet L., Vorobiev E., Grimi N., Mikhaylin S. (2020). Substantial Improvement of Tryptic and Chymotryptic Hydrolysis of β-Lactoglobulin Pretreated with High Voltage Electrical Treatments. ACS Sustain. Chem. Eng..

[42] Faucher M., Perreault V., Ciftci O.N., Gaaloul S., Bazinet L. (2021). Phospholipid Recovery from Sweet Whey and Whey Protein Concentrate: Use of Electrodialysis with Bipolar Membrane Combined with a Dilution Factor as an Ecoefficient Method. Future Foods.

[43] Laroche M., Perreault V., Marciniak A., Mikhaylin S., Doyen A. (2022). Eco-Efficiency of Mealworm (Tenebrio Molitor) Protein Extracts. ACS Food Sci. Technol..

[44] Ellen MacArthur Foundation Circular Economy Introduction. https://ellenmacarthurfoundation.org/topics/circular-economy-introduction/overview.

[45] What Is a Circular Economy? | Ellen MacArthur Foundation. https://ellenmacarthurfoundation.org/topics/circular-economy-introduction/overview.

[46] Ellen MacArthur Foundation The Butterfly Diagram: Visualising the Circular Economy. https://ellenmacarthurfoundation.org/circular-economy-diagram.

[47] Geissdoerfer M., Savaget P., Bocken N.M.P., Jan Hultink E. (2017). The Circular Economy—A New Sustainability Paradigm?. J. Clean. Prod..

[48] MacArthur E. (2013). Towards the Circular Economy. J. Ind. Ecol..

[49] Bocken N.M.P., Short S.W., Rana P., Evans S. (2014). A Literature and Practice Review to Develop Sustainable Business Model Archetypes. J. Clean. Prod..

[50] Weissbrod I., Bocken N.M.P. (2017). Developing Sustainable Business Experimentation Capability—A Case Study. J. Clean. Prod..

[51] Koseoglu-Imer D.Y., Karagunduz A. (2018). Recent Developments of Electromembrane Desalination Processes. Environ. Technol. Rev..

[52] Scarazzato T., Barros K.S., Benvenuti T., Rodrigues M.A.S., Espinosa D.C.R., Bernardes A.M.B., Amado F.D.R., Pérez-Herranz V., Basile A., Ghasemzadeh K. (2020). Chapter 5—Achievements in Electrodialysis Processes for Wastewater and Water Treatment. Current Trends and Future Developments on (Bio-) Membranes.

[53] Gurreri L., Tamburini A., Cipollina A., Micale G. (2020). Electrodialysis Applications in Wastewater Treatment for Environmental Protection and Resources Recovery: A Systematic Review on Progress and Perspectives. Membranes.

[54] Mir N., Bicer Y. (2021). Integration of Electrodialysis with Renewable Energy Sources for Sustainable Freshwater Production: A Review. J. Environ. Manage..

[55] Arana Juve J.-M., Christensen F.M.S., Wang Y., Wei Z. (2022). Electrodialysis for Metal Removal and Recovery: A Review. Chem. Eng. J..

[56] Maigrot E., Sabates J. (1890). Apparat Zur Lauterung von Zuckersaften Mittels Elektrizitat. German Patent.

[57] Cooney C.L., Humphrey A.E. (1985). The Principles of Biotechnology: Engineering Considerations.

[58] Campione A., Gurreri L., Ciofalo M., Micale G., Tamburini A., Cipollina A. (2018). Electrodialysis for Water Desalination: A Critical Assessment of Recent Developments on Process Fundamentals, Models and Applications. Desalination.

[59] Wilson J.R. (1960). Demineralization by Electrodialysis.

[60] Greiter M., Novalin S., Wendland M., Kulbe K.-D., Fischer J. (2002). Desalination of Whey by Electrodialysis and Ion Exchange Resins: Analysis of Both Processes with Regard to Sustainability by Calculating Their Cumulative Energy Demand. J. Membr. Sci..

[61] Gonçalves F., Fernandes C., Cameira dos Santos P., de Pinho M.N. (2003). Wine Tartaric Stabilization by Electrodialysis and Its Assessment by the Saturation Temperature. J. Food Eng..

[62] Vera E., Sandeaux J., Persin F., Pourcelly G., Dornier M., Ruales J. (2007). Deacidification of Clarified Tropical Fruit Juices by Electrodialysis. Part I. Influence of Operating Conditions on the Process Performances. J. Food Eng..

[63] Al-Amshawee S., Yunus M.Y.B.M., Azoddein A.A.M., Hassell D.G., Dakhil I.H., Hasan H.A. (2020). Electrodialysis Desalination for Water and Wastewater: A Review. Chem. Eng. J..

[64] Wang Y., Jiang C., Bazinet L., Xu T., Galanakis C.M. (2019). Chapter 10—Electrodialysis-Based Separation Technologies in the Food Industry. Separation of Functional Molecules in Food by Membrane Technology.

[65] Zhang Y., Paepen S., Pinoy L., Meesschaert B., Van der Bruggen B. (2012). Selectrodialysis: Fractionation of Divalent Ions from Monovalent Ions in a Novel Electrodialysis Stack. Sep. Purif. Technol..

[66] Huang C., Xu T. (2006). Electrodialysis with Bipolar Membranes for Sustainable Development. Environ. Sci. Technol..

[67] Frilette V.J. (1956). Preparation and Characterization of Bipolar Ion Exchange Membranes. J. Phys. Chem..

[68] Bazinet L., Castaigne F. (2019). Concepts de Génie Alimentaire - Procédés Associés, Application à La Conservation et Transformation Des Aliments.

[69] Tongwen X. (2002). Electrodialysis Processes with Bipolar Membranes (EDBM) in Environmental Protection—A Review. Resour. Conserv. Recycl..

[70] Pärnamäe R., Mareev S., Nikonenko V., Melnikov S., Sheldeshov N., Zabolotskii V., Hamelers H.V.M., Tedesco M. (2021). Bipolar Membranes: A Review on Principles, Latest Developments, and Applications. J. Membr. Sci..

[71] Nagasubramanian K., Chlanda F.P., Liu K.-J. (1977). Use of Bipolar Membranes for Generation of Acid and Base—An Engineering and Economic Analysis. J. Membr. Sci..

[72] Mani K.N., Chlanda F.P., Byszewski C.H. (1988). Aquatech Membrane Technology for Recovery of Acid/Base Values for Salt Streams. Desalination.

[73] Bazinet L., Lamarche F., Ippersiel D. (1998). Bipolar-Membrane Electrodialysis: Applications of Electrodialysis in the Food Industry. Trends Food Sci. Technol..

[74] Tronc J.-S., Lamarche F., Makhlouf J. (1997). Enzymatic Browning Inhibition in Cloudy Apple Juice by Electrodialysis. J. Food Sci..

[75] Bazinet L., Amiot J. (2008). Poulin, Jean-François; Tremblay, A.; Labbé, D. Process and System for Separation of Organic Charged Compounds. U.S. Patent.

[76] Henaux L., Thibodeau J., Pilon G., Gill T., Marette A., Bazinet L. (2019). How Charge and Triple Size-Selective Membrane Separation of Peptides from Salmon Protein Hydrolysate Orientate Their Biological Response on Glucose Uptake. Int. J. Mol. Sci..

[77] Doyen A., Saucier L., Beaulieu L., Pouliot Y., Bazinet L. (2012). Electroseparation of an Antibacterial Peptide Fraction from Snow Crab By-Products Hydrolysate by Electrodialysis with Ultrafiltration Membranes. Food Chem..

[78] Poulin J.-F., Amiot J., Bazinet L. (2006). Simultaneous Separation of Acid and Basic Bioactive Peptides by Electrodialysis with Ultrafiltration Membrane. J. Biotechnol..

[79] Firdaous L., Dhulster P., Amiot J., Gaudreau A., Lecouturier D., Kapel R., Lutin F., Vézina L.-P., Bazinet L. (2009). Concentration and Selective Separation of Bioactive Peptides from an Alfalfa White Protein Hydrolysate by Electrodialysis with Ultrafiltration Membranes. J. Membr. Sci..

[80] Roblet C., Akhtar M.J., Mikhaylin S., Pilon G., Gill T., Marette A., Bazinet L. (2016). Enhancement of Glucose Uptake in Muscular Cell by Peptide Fractions Separated by Electrodialysis with Filtration Membrane from Salmon Frame Protein Hydrolysate. J. Funct. Foods.

[81] Bazinet L., Moalic M. (2011). Coupling of Porous Filtration and Ion-Exchange Membranes in an Electrodialysis Stack and Impact on Cation Selectivity: A Novel Approach for Sea Water Demineralization and the Production of Physiological Water. Desalination.

[82] Lu H., Zou W., Chai P., Wang J., Bazinet L. (2016). Feasibility of Antibiotic and Sulfate Ions Separation from Wastewater Using Electrodialysis with Ultrafiltration Membrane. J. Clean. Prod..

[83] Doyen A., Husson E., Bazinet L. (2013). Use of an Electrodialytic Reactor for the Simultaneous β-Lactoglobulin Enzymatic Hydrolysis and Fractionation of Generated Bioactive Peptides. Food Chem..

[84] Suwal S., Rozoy É., Manenda M., Doyen A., Bazinet L. (2017). Comparative Study of in Situ and Ex Situ Enzymatic Hydrolysis of Milk Protein and Separation of Bioactive Peptides in an Electromembrane Reactor. ACS Sustain. Chem. Eng..

[85] Ndiaye N., Pouliot Y., Saucier L., Beaulieu L., Bazinet L. (2010). Electroseparation of Bovine Lactoferrin from Model and Whey Solutions. Sep. Purif. Technol..

[86] Aider M., Brunet S., Bazinet L. (2008). Electroseparation of Chitosan Oligomers by Electrodialysis with Ultrafiltration Membrane (EDUF) and Impact on Electrodialytic Parameters. J. Membr. Sci..

[87] Bazinet L., Cossec C., Gaudreau H., Desjardins Y. (2009). Production of a Phenolic Antioxidant Enriched Cranberry Juice by Electrodialysis with Filtration Membrane. J. Agric. Food Chem..

[88] Ge L., Wu B., Li Q., Wang Y., Yu D., Wu L., Pan J., Miao J., Xu T. (2016). Electrodialysis with Nanofiltration Membrane (EDNF) for High-Efficiency Cations Fractionation. J. Membr. Sci..

[89] Sheng F., Hou L., Wang X., Irfan M., Shehzad M.A., Wu B., Ren X., Ge L., Xu T. (2020). Electro-Nanofiltration Membranes with Positively Charged Polyamide Layer for Cations Separation. J. Membr. Sci..

[90] Zhao Z., Li X., Zhang H., Sheng F., Xu T., Zhu Y., Zhang H., Ge L., Xu T. (2022). Polyamide-Based Electronanofiltration Membranes for Efficient Anion Separation. Ind. Eng. Chem. Res..

[91] Dlask O., Václavíková N. (2018). Electrodialysis with Ultrafiltration Membranes for Peptide Separation. Chem. Pap..

[92] Sun L., Chen Q., Lu H., Wang J., Zhao J., Li P. (2020). Electrodialysis with Porous Membrane for Bioproduct Separation: Technology, Features, and Progress. Food Res. Int..

[93] Macedonio F., Curcio E., Drioli E. (2007). Integrated Membrane Systems for Seawater Desalination: Energetic and Exergetic Analysis, Economic Evaluation, Experimental Study. Desalination.

[94] Tufa R.A., Noviello Y., Di Profio G., Macedonio F., Ali A., Drioli E., Fontananova E., Bouzek K., Curcio E. (2019). Integrated Membrane Distillation-Reverse Electrodialysis System for Energy-Efficient Seawater Desalination. Appl. Energy.

[95] Tufa R.A., Pawlowski S., Veerman J., Bouzek K., Fontananova E., di Profio G., Velizarov S., Goulão Crespo J., Nijmeijer K., Curcio E. (2018). Progress and Prospects in Reverse Electrodialysis for Salinity Gradient Energy Conversion and Storage. Appl. Energy.

[96] Jiang C., Wang Y., Zhang Z., Xu T. (2014). Electrodialysis of Concentrated Brine from RO Plant to Produce Coarse Salt and Freshwater. J. Membr. Sci..

[97] Camacho L.M., Fox J.A., Ajedegba J.O. (2017). Optimization of Electrodialysis Metathesis (EDM) Desalination Using Factorial Design Methodology. Desalination.

[98] Alhéritière C., Ernst W.R., Davis T.A. (1998). Metathesis of Magnesium and Sodium Salt Systems by Electrodialysis. Desalination.

[99] Mohammadi R., Ramasamy D.L., Sillanpää M. (2021). Enhancement of Nitrate Removal and Recovery from Municipal Wastewater through Single- and Multi-Batch Electrodialysis: Process Optimisation and Energy Consumption. Desalination.

[100] Beery M., Wozny G., Repke J.-U., Pierucci S., Ferraris G.B. (2010). Sustainable Design of Different Seawater Reverse Osmosis Desalination Pretreatment Processes. Computer Aided Chemical Engineering.

[101] Beery M., Hortop A., Wozny G., Knops F., Repke J.-U. (2011). Carbon Footprint of Seawater Reverse Osmosis Desalination Pre-Treatment: Initial Results from a New Computational Tool. Desalination Water Treat..

[102] Tarnacki K.M., Melin T., Jansen A.E., van Medevoort J. (2011). Comparison of Environmental Impact and Energy Efficiency of Desalination Processes by LCA. Water Supply.

[103] Abou-Diab M., Thibodeau J., Deracinois B., Flahaut C., Fliss I., Dhulster P., Nedjar N., Bazinet L. (2020). Bovine Hemoglobin Enzymatic Hydrolysis by a New Ecoefficient Process—Part I: Feasibility of Electrodialysis with Bipolar Membrane and Production of Neokyotorphin (A137-141). Membranes.

[104] Abou-Diab M., Thibodeau J., Fliss I., Dhulster P., Nedjar N., Bazinet L. (2021). Eco-Circular Production of Demineralized Bioactive Peptides from Bovine Hemoglobin by Performing the Necessary Steps Simultaneously Using Bipolar Membrane Electrodialysis. ACS Sustain. Chem. Eng..

[105] Abou-Diab M., Thibodeau J., Deracinois B., Flahaut C., Fliss I., Dhulster P., Bazinet L., Nedjar N. (2020). Bovine Hemoglobin Enzymatic Hydrolysis by a New Eco-Efficient Process-Part II: Production of Bioactive Peptides. Membranes.

[106] Abou-Diab M., Thibodeau J., Fliss I., Dhulster P., Nedjar N., Bazinet L. (2021). Impact of Conductivity on the Performances of Electro-Acidification and Enzymatic Hydrolysis Phases of Bovine Hemoglobin by Electrodialysis with Bipolar Membranes for the Production of Bioactive Peptides. Sep. Purif. Technol..

[107] Pereira A.D., Gomide L.A.M., Cecon P.R., Fontes E.A.F., Fontes P.R., Ramos E.M., Vidigal J.G. (2014). Evaluation of Mortadella Formulated with Carbon Monoxide-Treated Porcine Blood. Meat Sci..

[108] Wei Y., Wang Y., Zhang X., Xu T. (2013). Comparative Study on Regenerating Sodium Hydroxide from the Spent Caustic by Bipolar Membrane Electrodialysis (BMED) and Electro-Electrodialysis (EED). Sep. Purif. Technol..

[109] Bazinet L., Lamarche F., Labrecque R., Toupin R., Boulet M., Ippersiel D. (1997). Electroacidification of Soybean Proteins for Production of Isolate. Food Technol. Chic..

[110] Alvarez F., Alvarez R., Coca J., Sandeaux J., Sandeaux R., Gavach C. (1997). Salicylic Acid Production by Electrodialysis with Bipolar Membranes. J. Membr. Sci..

[111] Liu X., Li Q., Jiang C., Lin X., Xu T. (2015). Bipolar Membrane Electrodialysis in Aqua–Ethanol Medium: Production of Salicylic Acid. J. Membr. Sci..

[112] Eisaman M., Alvarado L., Larner D., Wang P., Garg B., Littau K. (2011). CO_2_ Separation Using Bipolar Membrane Electrodialysis. Energy Environ. Sci..

[113] EPA Emission Facts: Average Carbon Dioxide Emissions Resulting from Gasoline and Diesel Fuel. https://nepis.epa.gov/Exe/ZyNET.exe/P1001YTF.TXT?ZyActionD=ZyDocument&Client=EPA&Index=2000+Thru+2005&Docs=&Query=&Time=&EndTime=&SearchMethod=1&TocRestrict=n&Toc=&TocEntry=&QField=&QFieldYear=&QFieldMonth=&QFieldDay=&IntQFieldOp=0&ExtQFieldOp=0&XmlQuery=&File=D%3A%5Czyfiles%5CIndex%20Data%5C00thru05%5CTxt%5C00000017%5CP1001YTF.txt&User=ANONYMOUS&Password=anonymous&SortMethod=h%7C-&MaximumDocuments=1&FuzzyDegree=0&ImageQuality=r75g8/r75g8/x150y150g16/i425&Display=hpfr&DefSeekPage=x&SearchBack=ZyActionL&Back=ZyActionS&BackDesc=Results%20page&MaximumPages=1&ZyEntry=1&SeekPage=x&ZyPURL.

[114] Eisaman M., Alvarado L., Larner D., Wang P., Littau K. (2011). CO_2_ Desorption Using High-Pressure Bipolar Membrane Electrodialysis. Energy Environ. Sci..

[115] Schaffner F., Pontalier P.-Y., Sanchez V., Lutin F. (2003). Bipolar Electrodialysis for Glycerin Production from Diester Wastes. Filtr. Sep..

[116] Herrero-Gonzalez M., Diaz-Guridi P., Dominguez-Ramos A., Irabien A., Ibañez R. (2020). Highly Concentrated HCl and NaOH from Brines Using Electrodialysis with Bipolar Membranes. Sep. Purif. Technol..

[117] Gazigil L., Er E., Kestioğlu O.E., Yonar T. (2022). Pilot-Scale Test Results of Electrodialysis Bipolar Membrane for Reverse-Osmosis Concentrate Recovery. Membranes.

[118] Aydin M.I., Yuzer B., Hasancebi B., Selcuk H. (2019). Application of Electrodialysis Membrane Process to Recovery Sulfuric Acid and Wastewater in the Chalcopyrite Mining Industry. Desalination Water Treat..

[119] Yuzer B., Aydin M.I., Yildiz H., Hasançebi B., Selcuk H., Kadmi Y. (2022). Optimal Performance of Electrodialysis Process for the Recovery of Acid Wastes in Wastewater: Practicing Circular Economy in Aluminum Finishing Industry. Chem. Eng. J..

[120] Paleologou M., Thibault A., Wong P.-Y., Thompson R., Berry R.M. (1997). Enhancement of the Current Efficiency for Sodium Hydroxide Production from Sodium Sulphate in a Two-Compartment Bipolar Membrane Electrodialysis System. Sep. Purif. Technol..

[121] Ghyselbrecht K., Huygebaert M., Van der Bruggen B., Ballet R., Meesschaert B., Pinoy L. (2013). Desalination of an Industrial Saline Water with Conventional and Bipolar Membrane Electrodialysis. Desalination.

[122] Reig M., Casas S., Valderrama C., Gibert O., Cortina J.L. (2016). Integration of Monopolar and Bipolar Electrodialysis for Valorization of Seawater Reverse Osmosis Desalination Brines: Production of Strong Acid and Base. Desalination.

[123] Herrero-Gonzalez M., Diaz-Guridi P., Dominguez-Ramos A., Ibañez R., Irabien A. (2018). Photovoltaic Solar Electrodialysis with Bipolar Membranes. Desalination.

[124] Sun Y., Wang Y., Peng Z., Liu Y. (2022). Treatment of High Salinity Sulfanilic Acid Wastewater by Bipolar Membrane Electrodialysis. Sep. Purif. Technol..

[125] Reig M., Valderrama C., Gibert O., Cortina J.L. (2016). Selectrodialysis and Bipolar Membrane Electrodialysis Combination for Industrial Process Brines Treatment: Monovalent-Divalent Ions Separation and Acid and Base Production. Desalination.

[126] Wei X., Wang Y., Yan H., Jiang C., Xu T. (2021). A Sustainable Valorization of Neopentyl Glycol Salt Waste Containing Sodium Formate via Bipolar Membrane Electrodialysis. Sep. Purif. Technol..

[127] Jiang C., Chen H., Zhang Y., Feng H., Shehzad M.A., Wang Y., Xu T. (2018). Complexation Electrodialysis as a General Method to Simultaneously Treat Wastewaters with Metal and Organic Matter. Chem. Eng. J..

[128] Wu X., Zhu H., Liu Y., Chen R., Qian Q., Van der Bruggen B. (2020). Cr(III) Recovery in Form of Na2CrO4 from Aqueous Solution Using Improved Bipolar Membrane Electrodialysis. J. Membr. Sci..

[129] Kimbrough D.E., Cohen Y., Winer A.M., Creelman L., Mabuni C. (1999). A Critical Assessment of Chromium in the Environment. Crit. Rev. Environ. Sci. Technol..

[130] Shi L., Hu Y., Xie S., Wu G., Hu Z., Zhan X. (2018). Recovery of Nutrients and Volatile Fatty Acids from Pig Manure Hydrolysate Using Two-Stage Bipolar Membrane Electrodialysis. Chem. Eng. J..

[131] Li C., Ramasamy D.L., Sillanpää M., Repo E. (2021). Separation and Concentration of Rare Earth Elements from Wastewater Using Electrodialysis Technology. Sep. Purif. Technol..

[132] Ramasamy D.L., Puhakka V., Doshi B., Iftekhar S., Sillanpää M. (2019). Fabrication of Carbon Nanotubes Reinforced Silica Composites with Improved Rare Earth Elements Adsorption Performance. Chem. Eng. J..

[133] Lin J., Chen Q., Huang X., Yan Z., Lin X., Ye W., Arcadio S., Luis P., Bi J., Van der Bruggen B. (2021). Integrated Loose Nanofiltration-Electrodialysis Process for Sustainable Resource Extraction from High-Salinity Textile Wastewater. J. Hazard. Mater..

[134] Benvenuti T., Siqueira Rodrigues M.A., Bernardes A.M., Zoppas-Ferreira J. (2017). Closing the Loop in the Electroplating Industry by Electrodialysis. J. Clean. Prod..

[135] Lopes A.C.A., Eda S.H., Andrade R.P., Amorim J.C., Duarte W.F., Grumezescu A.M., Holban A.M. (2019). 14—New Alcoholic Fermented Beverages—Potentials and Challenges. Fermented Beverages.

[136] Saffari M., Langrish T. (2014). Effect of Lactic Acid In-Process Crystallization of Lactose/Protein Powders during Spray Drying. J. Food Eng..

[137] Chandrapala J., Vasiljevic T. (2017). Properties of Spray Dried Lactose Powders Influenced by Presence of Lactic Acid and Calcium. J. Food Eng..

[138] Dufton G., Mikhaylin S., Gaaloul S., Bazinet L. (2019). Positive Impact of Pulsed Electric Field on Lactic Acid Removal, Demineralization and Membrane Scaling during Acid Whey Electrodialysis. Int. J. Mol. Sci..

[139] Talebi S., Garthe M., Roghmans F., Chen G.Q., Kentish S.E. (2021). Lactic Acid and Salt Separation Using Membrane Technology. Membranes.

[140] Talebi S., Suarez F., Chen G.Q., Chen X., Bathurst K., Kentish S.E. (2020). Pilot Study on the Removal of Lactic Acid and Minerals from Acid Whey Using Membrane Technology. ACS Sustain. Chem. Eng..

[141] Vecino X., Reig M., Gibert O., Valderrama C., Cortina J.L. (2020). Integration of Monopolar and Bipolar Electrodialysis Processes for Tartaric Acid Recovery from Residues of the Winery Industry. ACS Sustain. Chem. Eng..

[142] Wang Q., Chen G.Q., Lin L., Li X., Kentish S.E. (2021). Purification of Organic Acids Using Electrodialysis with Bipolar Membranes (EDBM) Combined with Monovalent Anion Selective Membranes. Sep. Purif. Technol..

[143] Wang Q., Chen G.Q., Kentish S.E. (2020). Isolation of Lactoferrin and Immunoglobulins from Dairy Whey by an Electrodialysis with Filtration Membrane Process. Sep. Purif. Technol..

[144] Kadel S., Thibodeau J., Parjikolaei B.R., Bazinet L. (2023). How PH Conditions Impact the Production of Growth Factors Enriched Fractions by Electro-Based Membrane Process.

[145] Faucher M., Geoffroy T.R., Thibodeau J., Gaaloul S., Bazinet L. (2022). Semi-Industrial Production of a DPP-IV and ACE Inhibitory Peptide Fraction from Whey Protein Concentrate Hydrolysate by Electrodialysis with Ultrafiltration Membrane. Membranes.

[146] Agriculture and Agri-Food Canada Red Meat and Livestock Slaughter Reports 2019. https://agriculture.canada.ca/en/sector/animal-industry/red-meat-and-livestock-market-information/slaughter-and-carcass-weights.

[147] Przybylski R., Firdaous L., Châtaigné G., Dhulster P., Nedjar N. (2016). Production of an Antimicrobial Peptide Derived from Slaughterhouse By-Product and Its Potential Application on Meat as Preservative. Food Chem..

[148] Bauer A.K., Dwyer-Nield L.D., Hankin J.A., Murphy R.C., Malkinson A.M. (2002). The Lung Tumor Promoter, Butylated Hydroxytoluene (BHT), Causes Chronic Inflammation in Promotion-Sensitive BALB/CByJ Mice but Not in Promotion-Resistant CXB4 Mice (Vol 169, Pg 1, 2001). Toxicology.

[149] Lanigan R.S., Yamarik T.A. (2002). Final Report on the Safety Assessment of BHT (1). Int. J. Toxicol..

[150] Przybylski R., Bazinet L., Firdaous L., Kouach M., Goossens J.-F., Dhulster P., Nedjar N. (2020). Harnessing Slaughterhouse By-Products: From Wastes to High-Added Value Natural Food Preservative. Food Chem..

[151] Przybylski R., Bazinet L., Firdaous L., Kouach M., Goossens J.-F., Dhulster P., Nedjar-Arroume N. (2020). Electroseparation of Slaughterhouse By-Product: Antimicrobial Peptide Enrichment by PH Modification. Membranes.

[152] Vanhoute M., Firdaous L., Bazinet L., Froidevaux R., Lecouturier D., Guillochon D., Dhulster P. (2010). Effect of Haem on the Fractionation of Bovine Haemoglobin Peptic Hydrolysate by Electrodialysis with Ultrafiltration Membranes. J. Membr. Sci..

[153] Sanchez-Reinoso Z., Cournoyer A., Thibodeau J., Said L.B., Fliss I., Bazinet L., Mikhaylin S. (2021). Effect of PH on the Antimicrobial Activity and Peptide Population of Pepsin Hydrolysates Derived from Bovine and Porcine Hemoglobins. ACS Food Sci. Technol..

[154] Cournoyer A., Thibodeau J., Ben Said L., Sanchez-Reinoso Z., Mikhaylin S., Fliss I., Bazinet L. (2022). How Discoloration of Porcine Cruor Hydrolysate Allowed the Identification of New Antifungal Peptides. Foods.

[155] Henaux L., Pereira K.D., Thibodeau J., Pilon G., Gill T., Marette A., Bazinet L. (2021). Glucoregulatory and Anti-Inflammatory Activities of Peptide Fractions Separated by Electrodialysis with Ultrafiltration Membranes from Salmon Protein Hydrolysate and Identification of Four Novel Glucoregulatory Peptides. Membranes.

[156] Renaud V., Houde V.P., Pilon G., Varin T.V., Roblet C., Marette A., Boutin Y., Bazinet L. (2021). The Concentration of Organic Acids in Cranberry Juice Modulates the Gut Microbiota in Mice. Int. J. Mol. Sci..

[157] McMurdo M.E.T., Bissett L.Y., Price R.J.G., Phillips G., Crombie I.K. (2005). Does Ingestion of Cranberry Juice Reduce Symptomatic Urinary Tract Infections in Older People in Hospital? A Double-Blind, Placebo-Controlled Trial. Age Ageing.

[158] Wing D.A., Rumney P.J., Preslicka C.W., Chung J.H. (2008). Daily Cranberry Juice for the Prevention of Asymptomatic Bacteriuria in Pregnancy: A Randomized, Controlled Pilot Study. J. Urol..

[159] Serre E., Boutin Y., Langevin M.-E., Lutin F., Pedneault K., Lacour S., Bazinet L. (2016). Deacidification of Cranberry Juice Protects against Disruption of In-Vitro Intestinal Cell Barrier Integrity. J. Funct. Foods.

[160] Renaud V., Faucher M., Perreault V., Serre E., Dubé P., Boutin Y., Bazinet L. (2020). Evolution of Cranberry Juice Compounds during in Vitro Digestion and Identification of the Organic Acid Responsible for the Disruption of in Vitro Intestinal Cell Barrier Integrity. J. Food Sci. Technol..

[161] Serre E., Rozoy E., Pedneault K., Lacour S., Bazinet L. (2016). Deacidification of Cranberry Juice by Electrodialysis: Impact of Membrane Types and Configurations on Acid Migration and Juice Physicochemical Characteristics (Vol 163, Pg 228, 2016). Sep. Purif. Technol..

[162] Shui G., Leong L.P. (2002). Separation and Determination of Organic Acids and Phenolic Compounds in Fruit Juices and Drinks by High-Performance Liquid Chromatography. J. Chromatogr. A.

[163] Flores P., Hellín P., Fenoll J. (2012). Determination of Organic Acids in Fruits and Vegetables by Liquid Chromatography with Tandem-Mass Spectrometry. Food Chem..

[164] Faucher M., Serre É., Langevin M.-È., Mikhaylin S., Lutin F., Bazinet L. (2018). Drastic Energy Consumption Reduction and Ecoefficiency Improvement of Cranberry Juice Deacidification by Electrodialysis with Bipolar Membranes at Semi-Industrial Scale: Reuse of the Recovery Solution. J. Membr. Sci..

[165] Akbas M.Y., Ölmez H. (2007). Inactivation of Escherichia Coli and Listeria Monocytogenes on Iceberg Lettuce by Dip Wash Treatments with Organic Acids. Lett. Appl. Microbiol..

[166] Castañer M., Gil M.I., Artes F., Tomas-Barberan F.A. (1996). Inhibition of Browning of Harvested Head Lettuce. J. Food Sci..

[167] Castañer M., Gil M.I., Artés F. (1997). Organic Acids as Browning Inhibitors on Harvested “Baby” Lettuce and Endive. Z. Leb. Forsch. A.

[168] Mullally M.M., Meisel H., FitzGerald R.J. (1997). Identification of a Novel Angiotensin-I-Converting Enzyme Inhibitory Peptide Corresponding to a Tryptic Fragment of Bovine β-Lactoglobulin. FEBS Lett..

[169] Nongonierma A.B., Mazzocchi C., Paolella S., FitzGerald R.J. (2017). Release of Dipeptidyl Peptidase IV (DPP-IV) Inhibitory Peptides from Milk Protein Isolate (MPI) during Enzymatic Hydrolysis. Food Res. Int..

[170] Morr C.V., Ha E.Y.W. (1991). Off-Flavors of Whey Protein Concentrates: A Literature Review. Int. Dairy J..

[171] Hwang D.-C., Damodaran S. (1995). Selective Precipitation and Removal of Lipids from Cheese Whey Using Chitosan. J. Agric. Food Chem..

[172] Damodaran S. (2011). Straightforward Process for Removal of Milk Fat Globule Membranes and Production of Fat-Free Whey Protein Concentrate from Cheese Whey. J. Agric. Food Chem..

[173] Adje E.Y., Balti R., Kouach M., Dhulster P., Guillochon D., Nedjar-Arroume N. (2011). Obtaining Antimicrobial Peptides by Controlled Peptic Hydrolysis of Bovine Hemoglobin. Int. J. Biol. Macromol..

[174] Küllenberg D., Taylor L.A., Schneider M., Massing U. (2012). Health Effects of Dietary Phospholipids. Lipids Health Dis..

[175] Pereira C.D., Diaz O., Cobos A. (2002). Valorization of By-Products from Ovine Cheese Manufacture: Clarification by Thermocalcic Precipitation/Microfiltration before Ultrafiltration. Int. Dairy J..

[176] Price N., Wan Z., Fei T., Clark S., Wang T. (2020). Development of Industrially Scalable Method for Phospholipids and Branch-Chain Fatty Acids of Dairy by-Product. J. Am. Oil Chem. Soc..

[177] Damodaran S. (2010). Zinc-Induced Precipitation of Milk Fat Globule Membranes: A Simple Method for the Preparation of Fat-Free Whey Protein Isolate. J. Agric. Food Chem..

[178] Gesan G., Daufin G., Merin U., Labbe J.-P., Quemerais A. (1995). Microfiltration Performance: Physicochemical Aspects of Whey Pretreatment. J. Dairy Res..

[179] Adolphson S.J., Ward L.S. (2016). Method for Defatting Whey Protein Concentrate and Producing Whey Protein Isolate. U.S. Patent.

[180] Chen G.Q., Eschbach F.I.I., Weeks M., Gras S.L., Kentish S.E. (2016). Removal of Lactic Acid from Acid Whey Using Electrodialysis. Sep. Purif. Technol..

[181] Dufton G., Mikhaylin S., Gaaloul S., Bazinet L. (2018). How Electrodialysis Configuration Influences Acid Whey Deacidification and Membrane Scaling. J. Dairy Sci..

[182] Dufton G., Mikhaylin S., Gaaloul S., Bazinet L. (2020). Systematic Study of the Impact of Pulsed Electric Field Parameters (Pulse/Pause Duration and Frequency) on ED Performances during Acid Whey Treatment. Membranes.

[183] Haddad M., Bazinet L., Barbeau B. (2021). Towards Water, Sodium Chloride and Natural Organic Matter Recovery from Ion Exchange Spent Brine. Membranes.

[184] Haddad M., Bazinet L., Barbeau B. (2019). Eco-Efficient Treatment of Ion Exchange Spent Brine via Electrodialysis to Recover NaCl and Minimize Waste Disposal. Sci. Total Environ..

[185] Oliveira V., Dias-Ferreira C., Labrincha J., Rocha J.L., Kirkelund G.M. (2020). Testing New Strategies to Improve the Recovery of Phosphorus from Anaerobically Digested Organic Fraction of Municipal Solid Waste. J. Chem. Technol. Biotechnol..

[186] Oliveira V., Kirkelund G.M., Horta C., Labrincha J., Dias-Ferreira C. (2019). Improving the Energy Efficiency of an Electrodialytic Process to Extract Phosphorus from Municipal Solid Waste Digestate through Different Strategies. Appl. Energy.

[187] Ebbers B., Ottosen L.M., Jensen P.E. (2015). Electrodialytic Treatment of Municipal Wastewater and Sludge for the Removal of Heavy Metals and Recovery of Phosphorus. Electrochimica Acta.

[188] Haddad M., Bazinet L., Savadogo O., Paris J. (2017). Electrochemical Acidification of Kraft Black Liquor: Impacts of Pulsed Electric Field Application on Bipolar Membrane Colloidal Fouling and Process Intensification. J. Membr. Sci..

[189] Zhu Y., Yan H., Lu F., Su Y., Li W., An J., Wang Y., Xu T. (2021). Electrodialytic Concentration of Landfill Leachate Effluent: Lab- and Pilot-Scale Test, and Assessment. Sep. Purif. Technol..

[190] Lejarazu-Larrañaga A., Molina S., Ortiz J.M., Navarro R., García-Calvo E. (2020). Circular Economy in Membrane Technology: Using End-of-Life Reverse Osmosis Modules for Preparation of Recycled Anion Exchange Membranes and Validation in Electrodialysis. J. Membr. Sci..

[191] Lejarazu-Larrañaga A., Molina S., Ortiz J.M., Riccardelli G., García-Calvo E. (2020). Influence of Acid/Base Activation Treatment in the Performance of Recycled Electromembrane for Fresh Water Production by Electrodialysis. Chemosphere.

